# Comparative Genomics of the Endosymbiont *Cardinium* Causing Reproductive Manipulation in *Encarsia* Parasitoid Wasps

**DOI:** 10.1002/mbo3.70084

**Published:** 2025-10-28

**Authors:** Dylan L. Schultz, Corinne M. Stouthamer, Suzanne E. Kelly, Olivia L. Mathieson, Manuel Kleiner, Martha S. Hunter, Stephan Schmitz‐Esser

**Affiliations:** ^1^ Department of Animal Science Iowa State University Ames Iowa USA; ^2^ Interdepartmental Microbiology Graduate Program Iowa State University Ames Iowa USA; ^3^ Department of Entomology University of Georgia Athens Georgia USA; ^4^ Department of Entomology The University of Arizona Tucson Arizona USA; ^5^ Department of Plant and Microbial Biology North Carolina State University Raleigh North Carolina USA; ^6^ Present Address: Department of Veterinary and Biomedical Sciences South Dakota State University Brookings South Dakota USA

**Keywords:** *Cardinium hertigii*, cytoplasmic incompatibility, horizontal gene transfer, metabolism, parasitoid wasp, parthenogenesis induction

## Abstract

Many invertebrates harbor the vertically transmitted endosymbiotic bacterium *Cardinium hertigii*, and some display altered reproductive phenotypes due to manipulation by *Cardinium*. Despite their host impact, genomic information for reproductive manipulator strains of *Cardinium* is sparse. Of the three reproductive manipulation phenotypes *Cardinium* is known to induce in its hosts, only two genomes causing cytoplasmic incompatibility (CI) are available, and genomes inducing other manipulation phenotypes are absent. In this study, we have sequenced and assembled four novel *Cardinium* genomes, three of which are associated with two different reproductive manipulation phenotypes, parthenogenesis induction and CI. Analysis of the genomes revealed that *Cardinium* associated with parasitoid wasp hosts in the genus *Encarsia* are generally more closely related to each other than to other *Cardinium*, but one strain, *c*Eina2, is very similar to the whitefly‐associated *Cardinium* strain *c*BtQ1. Further, unique and shared candidate genes for host interaction were identified, including putative zinc finger proteins shared by the parthenogenesis‐associated strains *c*Eper2 and *c*Ehis1 and a large protein encoded by the CI *Cardinium* strain *c*Eina3 with very distant similarity to the *Wolbachia* CI protein CidB. Finally, we predicted the presence of plasmids in three genomes. Also, despite the limited metabolic capacity of *Cardinium*, we identified potential horizontally transferred genes involved in central metabolism. These genomes will aid future studies to further our understanding of *Cardinium*‐induced reproductive manipulation.

## Graphical abstract

We assembled four *Cardinium hertigii* draft genomes: *c*Eina3 causes cytoplasmic incompatibility, *c*Ehis1 induces parthenogenesis, *c*Eper2 is associated with parthenogenesis, and *c*Eina2 infects asymptomatically. All four strains are closely related and hosted by *Encarsia* parasitoid wasps, but key differences exist, including their metabolic potential and how they may interact with hosts.

## 1 Introduction

Intracellular, maternally transmitted bacterial symbionts can exhibit an array of phenotypic impacts on their hosts, including nutritional benefits, defense, and reproductive manipulations (Kaltenpoth and Engl 2014; Doremus and Hunter 2020; Kaltenpoth et al. 2025). One such symbiont, *Cardinium hertigii*, a member of the *Bacteroidota* phylum, is estimated to infect 6%–13% of arthropod species (Zchori‐Fein and Perlman 2004; Russell et al. 2012; Weinert et al. 2015), and is especially prevalent in *Chelicerata* such as spiders and mites, as well as in insect orders *Hemiptera* and *Hymenoptera* (Duron et al. 2008; Russell et al. 2012; Weinert et al. 2015). Commonly known as a manipulator of its hosts' reproduction, *Cardinium* has been shown to induce parthenogenesis induction (PI), feminization, and cytoplasmic incompatibility (CI). It also may confer fitness benefits (Yang et al. 2021) and manipulate oviposition behavior (Kenyon and Hunter 2007). While *Cardinium* is a common symbiont that shares three of the four reproductive manipulations with the better‐known Alphaproteobacterial symbiont, *Wolbachia, Cardinium* biology, physiology, and genomes are understudied (Kaur et al. 2021; Mathieson et al. 2025). Just two *Cardinium* genomes implicated in CI are published, one from the white‐backed planthopper, *Sogatella furcifera* (Zeng et al. 2018), and another from the parasitoid wasp *Encarsia suzannae* (formerly *Encarsia pergandiella*) (Penz et al. 2012).

CI is the most common reproductive manipulation caused by reproductive manipulator symbionts. CI results in the death of offspring from crosses between a symbiont‐infected male and an uninfected female due to a symbiont‐mediated sabotage of male sperm. The lethal effect can be rescued, however, when the female also carries the symbiont. Best understood in *Wolbachia* where the CI‐causing genes were identified and named *cifs* (Beckmann et al. 2017; LePage et al. 2017), there are many parallels between CI caused by *Wolbachia* and *Cardinium*, including the cytological defects that result in early embryogenesis after a CI cross (Gebiola, Giorgini, et al. 2017; Hochstrasser 2023). However, no *cif* homologs have been identified in CI‐inducing *Cardinium* to date (Penz et al. 2012; Mann et al. 2017; Zeng et al. 2018). The best studied *Cardinium* CI system involves the parasitoid wasp host *E. suzannae* and the CI‐causing *c*Eper1 strain of *Cardinium*. The genome of *c*Eper1 was the first *Cardinium* genome sequenced (Penz et al. 2012), it was characterized by the first transcriptome (Mann et al. 2017) and it remains one of only two of over 20 *Cardinium* strains with both a known reproductive manipulation phenotype and a sequenced genome. This highlights a large gap in our knowledge of the genomics of reproductive manipulator strains of *Cardinium*.

Along with inducing CI in *E. suzannae, Cardinium* is also common in parthenogenetic (asexual) *Encarsia* wasps, where it is likely causing PI in most, if not all, cases (Zchori‐Fein et al. 2001). PI is a symbiont‐mediated asexual reproduction where, in haplodiploid hosts like *Encarsia*, haploid unfertilized eggs, which would normally develop as males, are instead converted into diploid eggs and become female offspring. A finding that antibiotic treatment causes *Cardinium‐*infected *Encarsia hispida* to produce diploid males indicates that two steps are required to make a parthenogenetic female by the symbiont: both diploidization and feminization (Giorgini et al. 2009). Despite the ecological importance and prevalence of PI, no genomes of parthenogenesis‐inducing *Cardinium* strains have been sequenced and the symbiont factors involved in producing this host phenotype remain unknown.

In this study, we present four closely related novel *Cardinium* genome assemblies from parasitoid wasp hosts in the genus *Encarsia*. Parasitoid wasps such as *Encarsia* are important for pest population control since they parasitize other insects by laying their eggs in or on suitable hosts, resulting in host death and successful maturation of the parasitoid offspring. *Encarsia* sp. have garnered considerable attention for their potential use in biological control of native and invasive crop pests such as the whitefly *Bemisia tabaci* (Hoddle et al. 1998; Liu et al. 2015) and are an important host group for studying *Cardinium*‐induced reproductive manipulation (Zchori‐Fein et al. 2001, 2004; Hunter et al. 2003; Gebiola et al. 2016; Doremus et al. 2022). Along with the aforementioned CI strain *c*Eper1 infecting *E. suzannae*, two *Encarsia‐*associated *Cardinium* genomes from this study are confirmed reproductive manipulators (PI and CI), while a third is associated with parthenogenesis, but causality cannot be established. The CI strain (*c*Eina3) infects its host at a very low density yet induces almost complete CI in its host, *Encarsia partenopea. Cardinium c*Eina2 coinfects *E. partenopea* with strain *c*Eina3, but *c*Eina2 does not induce a reproductive manipulation effect when infecting its host alone (Gebiola et al. 2016; Doremus et al. 2022). *Cardinium c*Ehis1 induces parthenogenesis in *E. hispida* (Zchori‐Fein et al. 2004). Finally, *c*Eper2 is associated with PI in its host, *Encarsia tabacivora*, but a causal link between this *Cardinium* strain and PI has not been established. The current community standard for linking PI to a symbiont relies on restoration of male offspring production through elimination of the symbiont via antibiotic treatment (Fricke and Lindsey 2024a). However, in the case of *c*Eper2, antibiotic treatment stops the wasp from reproducing entirely (Zchori‐Fein et al. 2001). Therefore, we describe *c*Eper2 as being “associated with PI” despite its status as the sole symbiont of a parthenogenetic host. With this study and the CI‐causing strain *c*Eper1, there are now five *Cardinium* genomes from strains infecting *Encarsia* parasitoid wasps, making *Encarsia* one of two host genera for which the most *Cardinium* genomic data exists, along with *Brevipalpus* mites. As *Cardinium* is an independent CI‐ and PI‐causing lineage distantly related to other known bacteria inducing these phenotypes, the availability of *Cardinium* genomes will allow a broader understanding of the host–symbiont interactions common to these phenomena. Here, we use previously and newly assembled *Cardinium* genomes from *Encarsia* hosts to draw comparisons among the genomes of this clade of symbionts causing varied reproductive manipulation phenotypes.

## 2 Materials and Methods

### 2.1 Wasp Cultures

The three hosts of the four *Cardinium* genomes include: (1) the parthenogenetic *E. tabacivora*, collected in Brazil and infected with *c*Eper2 (Zchori‐Fein et al. 2001), (2) *E. partenopea*, originally collected in Portici, Italy and coinfected with *c*Eina2 and the CI‐causing *c*Eina3 (White et al. 2009; Gebiola et al. 2016; Stouthamer et al. 2019), and (3) the parthenogenic *E. hispida*, originally collected in San Diego, CA, USA and infected with *c*Ehis1 (Zchori‐Fein et al. 2004). After a recent taxonomic revision, the nomenclature of two of three *Encarsia* bearing *Cardinium* was changed (Gebiola, Monti, et al. 2017). Both the parthenogen *E. tabacivora* and the host of the first CI *Cardinium* genome, *E. suzannae*, were known previously as *E. pergandiella* (Zchori‐Fein et al. 2001; Kenyon and Hunter 2007). In a separate study, *E. partenopea* was reclassified after it was found to be reproductively isolated from *Encarsia inaron* (Gebiola et al. 2016). All three *Encarsia* wasps that host the *Cardinium* of the current study are in laboratory culture in the Hunter laboratory in Tucson, AZ, USA, and were cultured on *B. tabaci* (strain MEAM1) nymphs infected with both *Rickettsia* and *Hamiltonella* but not *Cardinium* (Wang et al. 2019), on cowpea plants (*Vigna unguiculata*) at 27°C, ambient humidity. When the *Encarsia* wasps reached early pupation, the developing wasps were harvested and placed in a ventilated jar with honey and water to allow adult emergence. Adults were then collected for DNA extraction.

The *Cardinium* strains were sequenced using several platforms. Genomic DNA for long‐read Pacific Biosciences (PacBio, all strains) and Illumina MiSeq (*c*Eper2, *c*Eina2, and *c*Eina3) data sets was extracted using an enrichment technique for low‐density symbiont DNA from minute organisms as described previously (Stouthamer et al. 2018). Briefly, 1000 *Cardinium*‐infected *Encarsia* wasps (~ 19 mg) were homogenized in Buffer A (35 mM Tris–HCl, 250 mM sucrose, 250 mM ethylenediaminetetraacetic acid [EDTA], 25 mM KCl, 10 mM MgCl_2_) using a 1‐mL Dounce tissue grinder (Wheaton). The homogenate was incubated at 4°C for 1 h. The tissue debris was pelleted through centrifugation, and the supernatant was filtered through a 5‐µm pore size membrane. The suspension of enriched symbiont cells was pelleted through centrifugation, suspended in lysis buffer (0.5% sodium dodecyl sulfate, 200 mM Tris, 25 mM EDTA, 250 mM NaCl, and 1.3 mg/mL RNase A), and incubated at 37°C while shaking at 250 rpm for 30 min. Symbiont‐enriched DNA was extracted using phenol and chloroform, precipitated with 5 M NaCl and 100% ethanol, pelleted, and washed twice with 70% ethanol before being resuspended in TE buffer. PacBio sequencing was performed on an RS II machine at the University of Arizona Genomics Institute. Illumina MiSeq sequencing was performed by New York Medical College (New York City) following library preparation with a Nextera XT kit, producing 300 bp paired‐end reads with 400–600 bp insert size.

DNA for Illumina HiSeq libraries of *c*Ehis1, *c*Eina2, and *c*Eina3 was obtained as follows. Briefly, approximately 1000 wasps were ground with a 1‐mL Dounce tissue grinder (Wheaton) and extracted on a single column using the Qiagen DNeasy Blood and Tissue kit. Library preparation and sequencing were performed by the Vienna Biocenter Core Facilities VBCF NGS Unit (https://www.viennabiocenter.org/vbcf/) on an Illumina HiSeq 2500 sequencing platform with 150 bp paired‐end reads with 500–1000 bp inserts.

### 2.2 Genome Assembly

While each *Cardinium* genome was assembled using different assemblers due to the varying sequencing platforms used, our general strategy was to assemble the most *Cardinium*‐rich sequencing data set first and curate these contigs to obtain an initial assembly of *Cardinium*‐only contigs. We then mapped short‐read data to this curated assembly and extracted those reads to use for the final coassembly with long‐read sequencing data.

*Cardinium* strain *c*Ehis1 was sequenced using two different technologies: PacBio RS II and whole wasp Illumina HiSeq 2500 (2 × 150 bp). The Illumina reads were quality trimmed with Trimmomatic (settings: LEADING:3 TRAILING:3 SLIDINGWINDOW:4:20 MINLEN:36, v. 0.39) (Bolger et al. 2014). Using BLASTn (v. 2.2.26), PacBio bacterial subreads were identified and assembled using Flye (v. 2.9) using the PacBio‐raw and meta flags (Kolmogorov et al. 2019). The Illumina reads were mapped to this assembly using Bowtie2 (v. 2.4.1, setting: sensitive‐local) and indexed with Samtools (v. 1.14) (Langmead and Salzberg 2012; Danecek et al. 2021). The Flye assembly was error corrected in Pilon (v. 1.24) (Walker et al. 2014). Illumina reads, which mapped to the Flye assembly via Bowtie2 (v. 2.4.1, setting: sensitive‐local), were extracted and coassembled along with the *Cardinium* PacBio reads using Unicycler (v. 0.5.0, mode bold) (Wick et al. 2017). This resulted in an assembly of 42 contigs, 11 of which were identified as *Cardinium* based on their BLAST hits against NCBI's nonredundant (nr) protein database. These 11 *Cardinium* contigs comprise the draft *c*Ehis1 genome assembly.

*Cardinium* strain *c*Eper2 was sequenced using both PacBio RS II and MiSeq Nextera (2 × 300 bp). Symbiont‐enriched samples were used as input for the MiSeq data set (Stouthamer et al. 2018). The MiSeq reads were first quality trimmed in Trimmomatic (settings: LEADING:3 TRAILING:3 SLIDINGWINDOW:4:20 MINLEN:36, v. 0.39) and then merged in Pear (v. 0.9.11) (J. Zhang et al. 2014). PacBio reads were binned according to their BLASTn (v. 2.2.26) identification. The MiSeq reads and all *Cardinium*‐identified PacBio reads were assembled in SPAdes (v. 3.15.5) with a kmer size of 151 (Antipov et al. 2016). *Cardinium* contigs were identified using BlobTools (v. 1.1.1) and Bandage (v. 0.8.1) (Wick et al. 2015; Laetsch and Blaxter 2017). MiSeq reads were then mapped to the *Cardinium* contigs using Bowtie2 (v. 2.4.1, setting: sensitive‐local) and extracted from that assembly. These curated *Cardinium* PacBio and MiSeq reads were assembled in Unicycler (v. 0.5.0, mode bold) to generate the final *c*Eper2 draft assembly.

The coinfecting *Cardinium* strains *c*Eina2 and *c*Eina3 were broadly analyzed using the same strategy as the singly infected *Cardinium* genomes. The most *Cardinium*‐dense data sets were assembled, and contigs were curated as *Cardinium* or host. Then the short reads were mapped to this curated assembly, extracted, and assembled with the long‐read sequencing to produce the draft genome. We also included the additional step of sequencing singly infected wasps harboring *c*Eina2 to separate the coinfecting strains of *Cardinium*. *Cardinium* strains *c*Eina2 and *c*Eina3 coinfecting *E. partenopea* were sequenced with PacBio RS II and Illumina MiSeq Nextera (2 × 300 bp). The MiSeq reads were trimmed using Trimmomatic (settings: LEADING:3 TRAILING:3 SLIDINGWINDOW:4:20 MINLEN:36, v. 0.39) and merged using PEAR (v. 0.9.11). The PacBio reads were binned into symbiont and host reads using BLASTn (v. 2.2.26). MiSeq reads from coinfected *Encarsia* were used for an initial assembly (kmer = 227) using SPADes (v. 3.15.5). Contig clusters were visualized via Bandage (v.0.8.1), and the connected clusters that represented the coinfecting *c*Eina2 and *c*Eina3 genomes were kept. These contigs were filtered for erroneously placed host reads by keeping contigs identified as “*Cardinium*” by either dc‐megaBLAST or BLASTn (v. 2.2.26). The MiSeq reads were then mapped to these contigs using Bowtie (v. 2.4.1, setting: sensitive‐local). These mapped reads and filtered PacBio reads were assembled in SPAdes (kmer = 227) using the isolate flag. Contigs were removed from the resulting assembly if they met any of the following criteria: size below 300 bp, % guanine‐cytosine (GC) content above 42%, no BLASTn hit to *Cardinium*, or below 5× coverage as reported by SPAdes, resulting in an assembly of contigs belonging either to *c*Eina2 or *c*Eina3. To separate the high‐density *c*Eina2 genome and the low‐density *c*Eina3 genome, HiSeq sequencing data (150 bp PE) from *E. partenopea* infected only with *c*Eina2 were mapped to the combined *c*Eina2 and *c*Eina3 assembly. BlobTools (v. 1.1.1) was used to assess coverage of the contigs with the *c*Eina2‐only data set mapped. Contigs with coverage above 100 were categorized as *c*Eina2, but contigs with coverage between 60 and 350 were manually assessed, as repetitive elements shared by both genomes could skew coverage of contigs actually belonging to *c*Eina3. The coverage using *c*Eina2‐only reads of these contigs was visualized in Tablet (Milne et al. 2013) for uniformity‐mapped reads across the contig. Many displayed high coverage of reads mapping to a small region of the contig, which inflated the reported coverage despite very low coverage across the rest of the contig. Such contigs were assigned to *c*Eina3.

All final *Cardinium* genomes were uploaded to the Bacterial and Viral Bioinformatics Resource Center (BV‐BRC) website (Olson et al. 2023) for initial annotation and analysis for the purpose of this study, but final annotations were assigned by the NCBI Prokaryotic Gene Annotation Pipeline (PGAP) upon submission to the NCBI genomes database (BioProject accession: PRJNA1257813).

### 2.3 Plasmid Prediction

Potential plasmids were predicted by manual inspection of each draft genome. Genomic contigs over 5 Kbp in length with the following traits common to all known *Cardinium* plasmids were flagged as potential plasmids or pieces of potential plasmids (Penz et al. 2012; Santos‐Garcia et al. 2014; Xiong et al. 2023): (1) a reduced % GC content relative to the rest of the assembly, (2) the presence of putative plasmid partitioning proteins such as ParA/B (and more than 1 copy overall within the assembly), and (3) a lack of annotated housekeeping genes concurrent with an enrichment of unannotated hypothetical proteins and mobile genetic elements. Due to the fragmented nature of the assemblies, potential plasmids spanning multiple contigs were also considered, although they cannot be verified until more complete assemblies are available.

### 2.4 Assessment of Genome Coding Potential

Various tools were used to assess coding potential in the final assemblies. In brief, potential prophage regions were predicted by analyzing each genome with the Phastest webserver (Wishart et al. 2023). Secretion systems were predicted via BLASTp to components of the type VI secretion system (T6SS^IV^) and MacSyFinder (v. 2.1.3) using all available models (Néron et al. 2023). Metabolic and biosynthetic potential for each genome was predicted by the Ghost‐KOALA tool from the Kyoto Encyclopedia of Genes and Genomes database and manually compiled for analysis (Kanehisa et al. 2016). Average nucleotide identities (ANIs) were calculated using ANIb on the JSpecies webserver (Richter et al. 2016). Horizontally transmitted genes were predicted by HGTector (v. 2.0b3) (Zhu et al. 2014) followed by annotation/confirmation via BLASTp against the NCBI nr protein database. The “Proteome Comparison” tool on the BV‐BRC webserver (Olson et al. 2023) was used to conduct pairwise BLASTp searches between each *Encarsia‐*associated *Cardinium* genome to identify proteins that are shared or unique. Default settings were used for each tool unless otherwise noted.

### 2.5 Phylogenetic Trees

A whole‐genome phylogenetic tree was built with all available *Cardinium* genomes, *Amoebophilus asiaticus* (the closest known relative to *Cardinium*), and *Cytophaga hutchinsonii* within the *Cytophagales* order, as an outgroup, using the “Bacterial Genome Tree” tool on BV‐BRC with 188 genes, five duplications, and five deletions allowed (Olson et al. 2023). Briefly, amino acid and nucleotide sequences were aligned via MUSCLE (Edgar 2004) and the codon align function in BioPython (Cock et al. 2009), respectively. These alignments were used to generate a tree with 100 rounds of rapid bootstrapping via RAxML (Stamatakis et al. 2008; Stamatakis 2014) and visualized using the Archaeopteryx viewer on the BV‐BRC website (Olson et al. 2023). A single protein tree for pyruvate phosphate dikinase (PPDK) was built with MEGA12 (Kumar et al. 2024) using the Maximum Likelihood method and the Jones–Taylor–Thornton model of amino acid substitutions (Jones et al. 1992) with 1000 bootstrap replicates. Reference PPDK proteins were selected from BLASTp hits to the *Encarsia*‐associated *Cardinium* PPDK proteins along with PPDK proteins from organisms in diverse taxa.

## 3 Results and Discussion

### 3.1 Genome Assembly Information

Draft genomes were assembled for *C. hertigii* strains *c*Ehis1, *c*Eina2, *c*Eina3, and *c*Eper2, all of which are hosted by parasitoid wasps in the genus *Encarsia*. Due to the challenge of obtaining high‐quality, high molecular weight *Cardinium*‐enriched DNA from minute *Encarsia* wasps, our final assemblies were not closed with the available PacBio data. Additional long‐read sequencing will be required to close these genomes. Three of the sequenced strains are associated with reproductive manipulation in their respective host (*c*Eina3, *c*Ehis1, and *c*Eper2): *c*Eina3 causes CI (Doremus et al. 2022), *c*Ehis1 causes PI (Zchori‐Fein et al. 2004), *c*Eper2 is associated with PI (Zchori‐Fein et al. 2001), and *c*Eina2 infects asymptomatically (Doremus et al. 2022).

The genomes of *c*Ehis1, *c*Eina2, *c*Eina3, and *c*Eper2 were assembled into 11, 90, 82, and 86 contigs, respectively, using a combination of Illumina MiSeq, HiSeq, and PacBio sequencing data (Table 1). All novel genomes display a reduced % GC content (ranging from 35.8% to 36.6%), small assembly sizes between 0.86 and 1.08 Mbp, and 804–1046 coding sequences. The *c*Eina2, *c*Eina3, and *c*Ehis1 genome assemblies are similar to other published *Cardinium* genomes, with sizes around 1.0 Mbp and approximately 1000 encoded genes. Interestingly, *c*Eper2 has the smallest *Cardinium* genome sequenced to date at 0.86 Mbp and encodes the fewest number of predicted genes. This small size may be due to its status as a draft genome, but other larger *Cardinium* draft genomes contain a similar number or more contigs than *c*Eper2, making this an unlikely factor. A more plausible contributor is the lack of a predicted plasmid in *c*Eper2 (see below), since the chromosomes of *c*Eper2 and the closely related *c*Eper1 are very similar in size (0.86 Mbp in *c*Eper2 and 0.89 Mbp in *c*Eper1) while *c*Eper1 has an additional 57.8 Kbp plasmid.

**Table 1:** General assembly information for the genomes of *Encarsia*‐associated *Cardinium*.

*Cardinium* strain	*c*Eper2	*c*Ehis1	*c*Eina2	*c*Eina3	*c*Eper1
Host organism	*Encarsia tabacivora*	*Encarsia hispida*	*Encarsia partenopea*	*E. partenopea*	*Encarsia suzannae*
Manipulation phenotype	Associated with PI	PI	None	CI	CI
Sequencing approach	MiSeq, PacBio	HiSeq, PacBio	MiSeq, HiSeq, PacBio	MiSeq, HiSeq, PacBio	See (Penz et al. 2012)
Total assembly size (bp)	864,895	998,629	1,046,032	1,082,319	944,930
Total number of contigs	86	11	90	82	2 (chromosome + plasmid)
Contig N50	41,408	256,621	43,349	37,029	887,130
% GC	36.66	35.79	36.10	35.80	36.31
Total number of genes	804	858	1046	997	923
Number of plasmids	0	1 (predicted)	1 (predicted)	1 (predicted)	1
Plasmid size (bp)	N/A	124,732 (2 contigs)	Unknown	Unknown	57,800
Plasmid % GC	N/A	31.50	Unknown	Unknown	31.45
GenBank accession	GCA_050864935.1	GCA_050864915.1	GCA_050864895.1	GCA_050864855.1	GCA_000304455.1

Abbreviations: CI, cytoplasmic incompatibility; GC, guanine‐cytosine; PI, parthenogenesis induction.

### 3.2 Relatedness of *Encarsia*‐Associated *Cardinium* Strains

**Figure 1:**
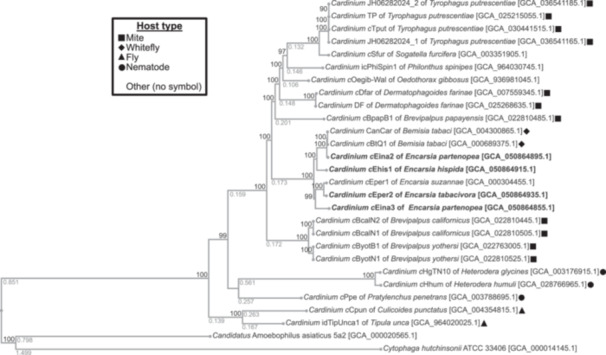
Phylogenetic tree of 26 currently available genomes classified as *Cardinium*. The tree was constructed using the “Bacterial Genome Tree” tool on the BV‐BRC website with 188 genes, five duplications, and five deletions allowed (Olson et al. 2023). Branch lengths above 0.1 are given below their respective branch, representing the estimated number of substitutions per site as an average of amino acid and nucleotide substitutions. Bootstrap confidence values of 90 or higher are shown above each node. Symbols indicate the type of host for each *Cardinium* strain, and NCBI GenBank accession numbers for each genome are given in brackets. BV‐BRC, Bacterial and Viral Bioinformatics Resource Center.

**Figure 2:**
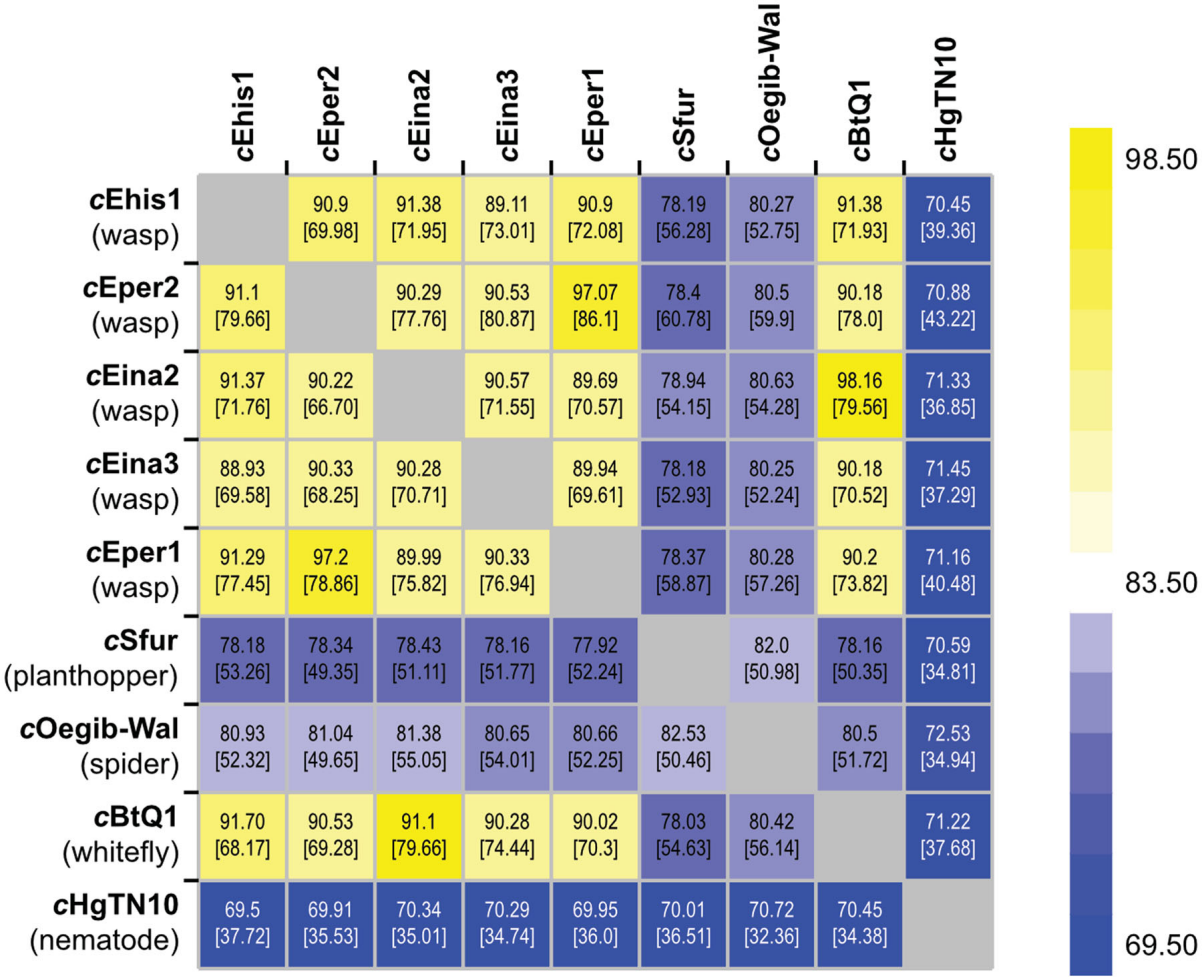
Average nucleotide identity and percentage overlap (shown in brackets) among *Encarsia*‐associated *Cardinium* and additional *Cardinium* genomes. Values were calculated via ANIb on the JSpecies webserver (Richter et al. 2016). Type of host is provided for ease of comparison. Genomes in the leftmost column were used as the reference genome for each comparison. ANI, average nucleotide identity.

A phylogenetic tree calculated with all currently available *Cardinium* genomes (*n* = 26) shows that *Encarsia*‐associated *Cardinium* are closely related. These strains form a clade with *Cardinium* infecting *B. tabaci* whiteflies (Figure 1). ANIs between the four newly assembled *Cardinium* genomes and other *Cardinium* genomes are shown in Figure 2. Strains *c*Ehis1, *c*Eina2, *c*Eina3, and *c*Eper2 share between 89% and 91.5% ANI with 70%–80% alignment overlap, suggesting there are sizeable differences in the content of the analyzed genomes even though all are hosted by parasitoid wasps of the same genus. The ANI to *Cardinium* strains found in arthropods and other insects was distinctly lower, ranging from approximately 70% to 80% ANI with alignment overlap percentages below 60%. We found that *c*Eper1 (hosted by *E. suzannae*) and *c*Eper2 (*E. tabacivora*) share an ANI above 97% (~ 80% alignment) and are much more closely related to each other than to the remaining *Encarsia*‐associated *Cardinium* and cluster together on the whole‐genome phylogenetic tree (Figure 1). It is possible that *c*Eper1 and *c*Eper2 diverged from a common ancestor relatively recently, as evidenced by their genome similarity. The two strains also infect highly related hosts which were previously both classified as *E. pergandiella*, but have since been revised into *E. suzannae* and *E. tabacivora* (Gebiola, Monti, et al. 2017). *Encarsia‐*associated *Cardinium* genomes also have varying degrees of synteny, with *c*Eper1 and *c*Eper2 encoding a highly similar genome structure while other genome pairs such *c*Eper1 and *c*Ehis1 are more structurally distant (Supporting Information File A1).

The phylogenetic analysis shown in Figure 1 places strain *c*Eina2 closer to *Cardinium* infecting whiteflies than to other *Encarsia*‐associated *Cardinium* strains, similar to what was seen previously (Stouthamer et al. 2019). Congruent with their close clustering in Figure 1, *Cardinium c*Eina2 and the *B. tabaci*‐infecting strain *c*BtQ1 share very high ANI (> 98% ANI, ~ 80% alignment) (Figure 2). The two genomes also have a high degree of synteny (Supporting Information File A1), further suggesting the genomes are very closely related. It is likely that the ancestor of *c*BtQ1 and *c*Eina2 was horizontally transmitted from *B. tabaci* to *Encarsia* or vice versa, potentially through the act of parasitism and development within *Bemisia* nymphs by *E. partenopea* larvae (Gebiola et al. 2016). Neither *c*Eina2 nor *c*BtQ1 is known reproductive manipulators in their respective hosts, and it is still unknown what phenotypic impacts *c*Eina2 may have on its host (Santos‐Garcia et al. 2014; Doremus et al. 2022). Therefore, there is a lingering question about how this asymptomatic symbiont has spread through populations of *E. partenopea*. One hypothesis is that the high‐density infection of *c*Eina2 is maternally transmitted with high efficiency. This may allow it to “hitch‐hike,” that is, benefit from CI caused by the coinfecting strain, *c*Eina3, while itself providing no discernible host fitness benefit or reproductive sabotage (Doremus et al. 2022). It is also possible that *c*Eina2 confers context‐dependent fitness benefits to *E. partenopea*, but none have yet been characterized. Uncovering the ways in which *c*Eina2 impacts its host and how the dynamics of *c*Eina2 and *c*Eina3 change in response to coinfection (instead of singly infecting a host) with regard to titer, gene expression, protein production, host phenotype, and so forth are intriguing topics for future research.

### 3.3 Putative Host Cell Interaction Genes

From evading the host immune system and manipulating reproduction to provisioning nutrients and providing defense against pathogens, intracellular symbiotic bacteria can interact with and influence their hosts in a variety of ways (Brownlie and Johnson 2009; Provorov and Onishchuk 2018; Doremus and Hunter 2020; Kaltenpoth et al. 2025). Although specific host‐manipulating effectors are not currently known for *Cardinium*, it is clear that this symbiont can induce changes in its hosts at the molecular and phenotypic levels, including and beyond reproductive manipulation (Mathieson et al. 2025). Secretion systems and their associated effectors likely play a crucial role in producing the phenotypes associated with *Cardinium*. For example, proteins with ankyrin repeat domains are found primarily in eukaryotes and are important for mediating protein‐protein interactions, which drive many aspects of cellular life (J. Li et al. 2006), but symbiont‐encoded ankyrin repeat proteins are believed to be crucially important effectors allowing symbionts to interact with and manipulate their hosts (Siozios et al. 2013). Host proteins, host chromatin, and the host ubiquitin system, which confers one of the most widespread and important eukaryotic posttranslational modifications (Damgaard 2021) have been posited as important targets for manipulation by symbionts. For example, CifB proteins responsible for causing CI by *Wolbachia* can contain deubiquitinating domains, peptidase domains, and nuclease domains (Tan et al. 2024). It is possible that proteins with similar domains may be important for *Cardinium*–host interactions, although other proteins are likely involved as well. In the following sections, we highlight some proteins encoded by *c*Ehis1, *c*Eina2, *c*Eina3, and *c*Eper2 that may be important for shaping the relationship between these symbionts and their hosts.

#### 3.3.1 Secretion Systems

Secretion systems are thought to be important tools for symbionts like *Cardinium* to establish successful associations with hosts, since a broad range of effectors for functions such as immune evasion and reproductive manipulation are likely required for key symbiont–host interactions. A common feature of all previously sequenced *Cardinium* genomes is the presence of a phage‐derived type VI secretion system (T6SS^IV^), which was first characterized in *A. asiaticus* (Böck et al. 2017). Indeed, *c*Ehis1, *c*Eina2, *c*Eina3, and *c*Eper2 also encode homologs of the T6SS^IV^ genes. This secretion system may play a key role in aspects of symbiosis such as host interaction or competition with other bacteria, although its substrates are currently unknown. *Cardinium c*Ehis1, *c*Eina2, and *c*Eina3 were also predicted to encode a complete type I secretion system (T1SS), and this secretion system may have been horizontally transferred between *Cardinium* and *Rickettsia* (discussed below). The T1SS is widespread in Gram‐negative bacteria and is used to secrete a variety of proteins, including toxins and other effectors, outside of the cell (Spitz et al. 2019). However, the functionality and substrates of this secretion system in *Cardinium* have not been explored. A type IX secretion system (T9SS), which is exclusive to the *Bacteroidota* phylum and is important for gliding motility and the secretion of virulence factors (Gorasia et al. 2020), was not predicted for any *Encarsia*‐associated *Cardinium* genome.

#### 3.3.2 The Presence of Highly Transcribed *c*Eper1 Genes in Other *Encarsia*‐Associated *Cardinium*

With no additional molecular data available for *c*Ehis1, *c*Eina2, *c*Eina3, and *c*Eper2, it is not currently possible to ascertain which genes are highly transcribed and may be important for host interaction or manipulation by these *Cardinium* strains. We used transcriptomic data available for *c*Eper1 (Mann et al. 2017) to speculate on host interaction candidate genes by identifying homologs to genes that were highly transcribed in *c*Eper1. As shown in Table 2, many highly transcribed nonhousekeeping genes encoded by *c*Eper1 have homologs in other *Encarsia‐*associated *Cardinium* genomes. Such homologs include outer membrane proteins potentially involved in processes like adhesion, evasion, and molecule transport (Fairman et al. 2011), lipoproteins, and homologs very similar to a *c*Eper1 DEAD box RNA helicase (> 96% amino acid identity). DEAD box RNA helicase genes are involved in RNA metabolism, including recognizing and binding foreign nucleic acids and inducing intracellular immune responses (Taschuk and Cherry 2020). CAHE_0662, another highly transcribed and uncharacterized *c*Eper1 gene, has high amino acid identity (> 70%) with full‐length homologs in all *Encarsia*‐associated *Cardinium* and was previously hypothesized to be involved with inhibiting apoptosis of infected host cells (Mann et al. 2017). Of the four highly transcribed plasmid‐encoded *c*Eper1 genes, only CAHE_p0026 and CAHE_p0043 are not conserved in all five *Encarsia*‐associated *Cardinium* genomes. CAHE_p0026, which has a low‐identity homolog only in *c*Ehis1, was identified as a potential candidate for involvement in host interaction or *c*Eper1 CI due to its predicted ankyrin repeats and RING‐like domain, which may confer ubiquitin ligase activity (Penz et al. 2012; Mann et al. 2017). Additionally, the predicted lipoprotein CAHE_p0043 is only shared between the CI‐causing *Cardinium* strains *c*Eper1 and *c*Eina3, although its potential function is unknown. It is important to recognize that high transcription levels of genes in one organism do not indicate that homologs in other organisms will be similarly expressed. Even so, the potential role of these *c*Eper1 proteins and their homologs in host interaction should be explored, as sequence conservation of highly transcribed *c*Eper1 genes across *Encarsia‐*associated *Cardinium* suggests these candidates may be important for *Cardinium*‐*Encarsia* symbioses.

**Table 2:** Similarity of highly transcribed potential host‐interacting proteins from *c*Eper1 in other *Encarsia*‐associated *Cardinium* strains.

*c*Eper1 protein locus tag|UniProt accession	UniProt annotation of *c*Eper1 protein	% ID in *c*Ehis1	% ID in *c*Eina2	% ID in *c*Eina3	% ID in *c*Eper2
CAHE_0050|K0NZZ8	Outer membrane protein beta‐barrel domain‐containing protein	77.38	62.04	68.87	64.14
CAHE_0105|K0P009	Outer membrane protein beta‐barrel domain‐containing protein	58.20	75.00	71.20	61.40
CAHE_0390|K0P065	Outer membrane protein beta‐barrel domain‐containing protein	74.10	71.46	77.25	74.38
CAHE_0406|K0P5U4	Lipoprotein	58.62	61.67	64.29	90.00
CAHE_0435|K0P074	Ankyrin repeat	68.53	56.64	72.73	74.94
CAHE_0662|K0P2G8	Uncharacterized protein RP147	78.86	85.08	71.95	76.02
CAHE_0677|K0P2H4	DEAD box RNA helicase	96.92	96.92	96.14	97.17
CAHE_0757|K0P2K4	Lipoprotein	28.61	42.23	36.61	28.00
CAHE_p0007|K0PB04	Uncharacterized protein	53.07	35.21	35.99	86.91
CAHE_p0014|K0P0F9	Uncharacterized protein	47.27	34.41	34.58	83.02
CAHE_p0026|K0P2Q1	RING domain‐containing protein	25.95	0.00	0.00	0.00
CAHE_p0043|K0P6Z1	Lipoprotein	0.00	0.00	38.02	0.00

*Note:* Values shown in the “% ID” columns are the percent amino acid identities of the best full‐length (or nearly full‐length) hit to the given *c*Eper1 protein from each genome. Proteins denoted with a “p” in the locus tag are encoded on the *c*Eper1 plasmid, pCher. Accession numbers for *c*Ehis1, *c*Eina2, *c*Eina3, and *c*Eper2 proteins similar to highly transcribed *c*Eper1 proteins are given in Supporting Information Table A1.

#### 3.3.3 Comparison of CI‐Causing *Cardinium*

*Cardinium* strains *c*Eper1 and *c*Eina3 appear to be fairly distantly related with less than 90% ANI (Figure 2), but have both been shown to cause CI, or embryonic death resulting from crosses between uninfected females and *Cardinium*‐infected males, in their respective hosts. The *Cardinium* effectors that cause and rescue CI are currently unknown (Hunter et al. 2003; Gebiola et al. 2016). Previous research suggested that *c*Eper1 and *c*Eina3 may induce CI using separate methods and/or by different genes due to their stark differences in localization pattern and infection density within their hosts (Doremus et al. 2020, 2022). Strain *c*Eper1 infects developing sperm cells, giving it a chance to directly prime sperm cells for causing CI. Strain *c*Eina3 does not infect sperm cells, instead localizing to somatic cells in the testis and seminal vesicle, suggesting it may prime sperm for CI through more indirect means (Doremus et al. 2020, 2022). *c*Eina3 is also at very low overall abundance in its host compared with *c*Eper1, but CI caused by *c*Eina3 is more uniformly lethal than CI caused by *c*Eper1 (nearly 100% mortality for *c*Eina3 vs. 80%–90% mortality for *c*Eper1) (Gebiola et al. 2016; Doremus et al. 2022).

Although it is tempting to speculate that homologous genes shared between CI‐causing *Encarsia‐*associated *Cardinium* strains with characteristics of host‐interacting proteins may be involved in causing CI, we refrain from classifying them as CI candidates because *Cardinium c*Eper1 and *c*Eina3 may use different mechanisms for causing CI (Doremus et al. 2020, 2022). Nevertheless, such genes are intriguing candidates for regulating host interaction and merit further study. For example, *c*Eper1 CAHE_0286 and cEina3 MGI2299016.1 are homologous patatin‐like proteins not found in other *Cardinium* genomes and with the closest similar proteins encoded by *Wolbachia*. Patatins are phospholipases important for bacterial virulence, including causing host cell lysis, protection against host endosome formation, and aiding escape and spread through host cells by organisms such as *Pseudomonas aeruginosa*, *Rickettsia*, and *Legionella pneumophila* (Sato 2003; Aurass et al. 2006; Rahman et al. 2013; Gaspar and Machner 2014; Borgo et al. 2022), although their function in *Cardinium* is unknown. Strains *c*Eper1 (CAHE_0706) and *c*Eina3 (MGI2298593.1) also encode homologous proteins containing collagen triple helix repeats, which are not found in other *Encarsia*‐associated *Cardinium*. These proteins may be involved in cell structure, host cell invasion, or evading the host immune system (Yu et al. 2014).

Additionally, the *c*Eina3 genome encodes two adjacent proteins, MGI2299066.1 and MGI2299067.1, which are homologs of the adjacent *c*Eper1 pCher plasmid‐encoded genes CAHE_p0043 (a predicted lipoprotein) and CAHE_p0044 (Knr4/Smi1‐like domain‐containing protein). Interestingly, MGI2299067.1 has a distant homolog within the *c*Eina3 genome, MGI2298375.1, but it is unclear if they are plasmid or chromosomally encoded in the draft genome assembly. This potential duplication event seems to be conserved in the *c*Eper1 genome, where CAHE_p0044 has a chromosomally encoded similar protein (CAHE_0817; 26.96% amino acid identity). Like CAHE_p0044, MGI2299067.1, MGI2298375.1, and CAHE_0817 have predicted Knr4/Smi1‐like domains. Knr4/Smi1 proteins were initially characterized as being involved with regulating fungal cell wall assembly and cell cycle progression while protecting against cell wall stressors (Hong et al. 1994; Kroll et al. 2025), but members of this protein family have also been hypothesized to be antitoxins or immunity proteins for a range of toxins (D. Zhang et al. 2011). Although it is unclear what function *Cardinium*‐encoded Knr4/Smi1 homologs may have, they could be involved broadly in host interaction. Additional experimental work is required to identify and test *Cardinium* genes for potential roles in CI and host manipulation.

#### 3.3.4 Comparison of PI *Cardinium* and Other PI Bacteria

*Cardinium* strains *c*Eper2 and *c*Ehis1 are both associated with asexual parthenogenetic female hosts (Zchori‐Fein et al. 2001; Zchori‐Fein et al. 2004), with only *c*Ehis1 being causally linked to PI induction through restoring the production of males via curing the host of the symbiont (Zchori‐Fein et al. 2004). While the mechanisms for *Cardinium*‐induced PI are largely unknown, it has been shown that diploidy restoration in unfertilized eggs likely plays a role along with the activation of relevant genes in the host sex‐determination cascade (Giorgini 2007; Giorgini et al. 2009; Doremus and Hunter 2020). Specific *Cardinium* genes associated with host sex determination or diploidy restoration have yet to be identified, though they likely involve interaction with chromatin. Candidates for PI‐inducing factors have been identified in some strains of PI‐causing *Wolbachia*: *pifA* and *pifB* encoded by *Wolbachia w*Tpre infecting the parasitoid wasp *Trichogramma pretiosum* (Fricke and Lindsey 2024b), and *piff*, which is similar to *pifA*, in *Wolbachia* infecting *Encarsia formosa* (C. Li et al. 2024). Both PifA and Piff share similarity with the insect sex‐determination gene *transformer (tra)*. It was proposed that symbiont‐encoded PifA/Piff functions as mimics of the host female‐specific Tra, sparking the downstream female sex‐determination cascade to induce PI (Fricke and Lindsey 2024b; C. Li et al. 2024), further suggesting that PI induction may rely on symbiont‐derived regulation of host sex‐determination pathways.

BLASTp searches of PifA and PifB from *Wolbachia* strain *w*Tpre (Fricke and Lindsey 2024b), and Transformer from *E. suzannae* (Schultz et al. 2022) against proteins encoded by *c*Eper2 and *c*Ehis1 yielded no hits with an *e*‐value < 0.05, suggesting that these *Cardinium* strains likely induce PI using different factors than those recently identified in *Wolbachia*. One set of potential candidate genes for PI induction by *c*Eper2 and *c*Ehis1 encodes homologous putative zinc finger proteins (*c*Eper2 MGI2262406.1 and *c*Ehis1 MGI2257526.1) found in the PI‐associated strains and absent from other *Encarsia*‐associated *Cardinium*. The *c*Eper2 and *c*Ehis1 zinc finger proteins are 86 and 203 amino acids long, respectively, and the best BLASTp hits for both proteins include zinc finger proteins from a range of eukaryotes, including aphids and fish, with hits to bacterial zinc finger proteins being of lower quality. Zinc finger proteins are common in eukaryotes and are broadly involved in binding DNA, meaning they can have impacts on a wide array of cellular processes, including gene regulation (Malgieri et al. 2015). Interestingly, *doublesex* and *fruitless*, genes central to sex determination and sexual behavior in many insects and previously found to be expressed by *E. suzannae* (Schultz et al. 2022), also encode zinc finger domains to provide DNA‐binding properties (Erdman and Burtis 1993; Ito et al. 1996; Bertossa et al. 2009). The *c*Eper2 and *c*Ehis1 zinc finger proteins have distant similarity to *E. suzannae* Fruitless proteins (33.3% amino acid identity with 3e^−05^ *e*‐value and 35.09% identity with 0.003 e‐value, respectively), but do not share homology with *E. suzannae doublesex* or other genes in the sex‐determination cascade. It is still unclear what role the zinc finger proteins unique to *c*Eper2 and *c*Ehis1 may have in shaping the association between *c*Eper2, *c*Ehis1, and their parthenogenetic hosts. These proteins are interesting candidates for future study due to their similarity to eukaryotic zinc finger proteins and their potential for interaction with host DNA, perhaps as *Cardinium*‐encoded transcriptional regulators of host genes or as PI effectors.

#### 3.3.5 Proteins Unique Among *Encarsia*‐Associated *Cardinium*

Pairwise BLASTp searches of *Encarsia*‐associated *Cardinium* genomes (*c*Eper1, *c*Eper2, *c*Ehis1, *c*Eina2, and *c*Eina3) revealed many proteins that are unique to each *Cardinium* genome when compared against all other *Encarsia*‐associated *Cardinium*. There are 21 proteins larger than 100 amino acids unique to *c*Ehis1, 22 unique to *c*Eina2, 45 unique to *c*Eina3, 15 unique to *c*Eper2, and 13 unique to *c*Eper1. Some of these proteins might be involved in host interaction, such as through siphoning host resources, interacting with host DNA or RNA, cleaving proteins, and other unknown mechanisms.

Unique proteins larger than 300 amino acids were subject to BLASTp (*e*‐value < 1e^−5^) against the NCBI nr protein database to gain additional information on protein annotation. Proteins with potential for host interaction are shown in Table 3. These include a protein encoded by *c*Ehis1 similar to a DEAD/DEAH box helicase (MGI2257498.1), proteins encoded by *c*Ehis1 and *c*Eina2 with homology to ankyrin repeat domain‐containing proteins (MGI2257497.1 and MGI2299977.1), and a protein encoded by *c*Eper2 with distant similarity to a type 2 lanthipeptide synthetase (MGI2262336.1). Lanthipeptides are mainly known for their antibiotic properties (also called lantibiotics), but they can also have antifungal or antiviral properties, among others (Repka et al. 2017). Previous studies have shown that *Cardinium* infection can negatively influence other symbionts and the diversity of host microbiota, but the mechanisms are unknown (T.‐P. Li et al. 2020, 2022; Nesvorna et al. 2020; Hubert et al. 2021, 2025). Perhaps *c*Eper2 generates defensive lanthipeptides to compete with other bacteria and protect itself or its host from potential pathogens.

**Table 3:** List of selected proteins larger than 300 amino acids unique to one of the five *Encarsia*‐associated *Cardinium* genomes when compared against each other. BLASTp was conducted using the standard NCBI nonredundant protein database and a minimum e‐value of 1e^−4^.

*Cardinium* strain and GenBank protein accession	Protein length (AA)	BLASTp hit	Amino acid identity (%)	Query coverage (%)
*c*Ehis1 MGI2257498.1	496	MEL6152539.1: DEAD/DEAH box helicase (*Bacteroidota* bacterium)	34.93	97
		WP_083758808.1: Ankyrin repeat domain‐containing protein (*Candidatus Amoebophilus asiaticus*)	44.78	66
*c*Ehis1 MGI2257497.1	493	WP_353285904.1: Ankyrin repeat domain‐containing protein (*Wolbachia* endosymbiont of *Rhorus exstirpatorius*)	29.05	29
*c*Eina2 MGI2299977.1	430	WP_034576254.1: Ankyrin repeat domain‐containing protein (*Cardinium* endosymbiont of *Bemisia tabaci*)	98.87	100
*c*Eper2 MGI2262336.1	556	MGB9911660.1: Type 2 lanthipeptide synthetase LanM (Microgenomates group bacterium)	27.59	91
*c*Eina3 MGI2298700.1	1535	WP_157752277.1: PD‐(D/E)XK nuclease domain‐containing protein (*Rickettsia amblyommatis*)	29.05	21
		MFJ5423312.1: CifB (*Wolbachia* endosymbiont of *Drosophila barbarae*)	32.08	7
*c*Eina3 MGI2298912.1	538	WP_025424378.1: S8 family peptidase (*Sodalis praecaptivus*)	58.21	99
*c*Eina3 MGI2298419.1	367	WP_243575259.1: SET domain‐containing protein (*Cardinium c*ByotN1)	37.25	95
*c*Eina3 MGI2298253.1	339	MBA8667100.1: Dicarboxylate/amino acid:cation symporter (*Holosporaceae* bacterium)	53.89	97

Strain *c*Eina3 also encodes many unique proteins, including an S8 family serine peptidase, a dicarboxylate/amino acid‐cation symporter which may allow for the import of host‐derived amino acids, and a SET domain‐containing protein which can use *S*‐adenosyl‐l‐methionine (SAM) as a cofactor to methylate substrates, such as histones (Herz et al. 2013). *c*Eina3 also encodes a homolog of an SAM transporter characterized in *A. asiaticus* (48% amino acid identity, MGI2298647.1; *A. asiaticus*: ACP21098.1) (Haferkamp et al. 2013), potentially allowing it to take up SAM as a cofactor for methylation reactions without needing to synthesize SAM directly. It is possible that the SET domain‐containing protein may be involved in altering host cell gene regulation or chromatin states, leading to varying impacts on host cell biology (Greer and Shi 2012). Another *c*Eina3 protein with potential for host interaction is a 1535 amino acid protein (MGI2298700.1) with ankyrin repeat domains, similarity to PD‐(D/E)XK nuclease domain‐containing proteins, which is a domain encoded also by many *Wolbachia* CifB proteins (Shropshire et al. 2020), and a small region with similarity to *Wolbachia* CifB. The short alignment length (7% coverage) and identity of the BLASTp hits to CifB (Table 3), combined with a lack of hits to CifB proteins via HHPred (Zimmermann et al. 2018; Gabler et al. 2020) and UniProt (Bateman et al. 2025) searches, suggest this protein may not be a homolog of CifB. Further, it is unknown if there is an adjacent protein similar to CifA since MGI2298700.1 is encoded on a small contig along with only transposases. Nevertheless, future work is warranted to identify whether these proteins are involved in CI or other aspects of host interaction by *c*Eina3 and other *Encarsia*‐associated *Cardinium*.

### 3.4 Mobile Genetic Elements

Mobile genetic elements are common in the newly assembled *Cardinium* genomes and include predicted plasmids and many transposable elements (TEs). However, there is little evidence for complete prophages in any of the four genomes. *Cardinium* strains *c*Ehis1, *c*Eina2, *c*Eina3, and *c*Eper2 appear to have many TEs, with 28, 159, 82, and 41 transposase enzymes encoded by each genome, respectively, as annotated by PGAP.

Plasmids are likely underrepresented in published *Cardinium* genome assemblies due to the few available complete genomes, but strains *c*Eper1, *c*BtQ1, and DF were predicted to contain one (*c*Eper1 and *c*BtQ1) or two (DF) plasmids (Penz et al. 2012; Santos‐Garcia et al. 2014; Xiong et al. 2023). Of the newly assembled genomes, all except *c*Eper2 were predicted to potentially encode a plasmid based on the presence of contigs with features similar to other known *Cardinium* plasmids (Penz et al. 2012; Santos‐Garcia et al. 2014; Xiong et al. 2023). The fragmented nature of the *c*Eina2 and *c*Eina3 assemblies caused potential plasmids to be split across multiple contigs, but contigs with plasmid‐like features such as JBOZWX010000020.1 (14.5 Kbp), JBOZWX010000031.1 (12.3 Kbp), and JBOZWX010000014.1 (10.8 Kbp) in *c*Eina2 and JBOZWW010000020.1 (16.8 Kbp), JBOZWW010000001.1 (14.6 Kbp), and JBOZWW010000006.1 (13.4 Kbp) in *c*Eina3 were identified. Therefore, we predict that plasmids exist in those genomes; however, due to the fragmented nature of these assemblies, we cannot currently predict plasmid sizes in *c*Eina2 and *c*Eina3.

*Cardinium c*Ehis1 is predicted to encode a 125‐Kbp plasmid assembled into two contigs. Contig 4 (JBOZWY010000004.1) is a predicted component of the plasmid since it matches the expected size (116 Kbp), % GC content (31.52%), and coding potential for a *Cardinium* plasmid: it encodes no bacterial housekeeping genes besides three chromosome/plasmid partitioning genes (including *parA* and *parB*). Further, *c*Ehis1 contig 10 (JBOZWY010000010.1) also exhibits characteristics of a partial *Cardinium* plasmid at 8.6 Kbp and 30.96% GC while encoding only hypothetical proteins and a guanosine‐3′,5′‐bis(diphosphate) 3′‐pyrophosphohydrolase (but no *parA* or *parB*). BLASTn comparisons revealed that contigs 4 and 10 from *c*Ehis1 also show high similarity (77.7%–93.3% nucleotide identity with coverage varying from 9% to 62%) to known *Cardinium* plasmids from strains DF, *c*Eper1, and *c*BtQ1 (Penz et al. 2012; Santos‐Garcia et al. 2014; Xiong et al. 2023). The *c*Ehis1 genome assembly also encodes homologs to nearly all proteins from the *c*Eper1 plasmid pCher (Supporting Information Figure A1), suggesting that *c*Ehis1 contains a plasmid similar to pCher. Additional sequencing to generate closed genomes of these strains will be required to confirm the presence and size of putative plasmids.

### 3.5 Horizontally Transferred Genes

Obligate intracellular symbionts have limited opportunities for interaction with other bacteria due to their lifestyle, but their hosts are frequently coinfected by multiple distinct bacterial symbionts (Duron et al. 2008; McLean et al. 2018). Therefore, other symbiotic bacteria are the most likely partners for horizontal gene transfer (HGT). There were 107 (*c*Ehis1), 201 (*c*Eina2), 109 (*c*Eina3), and 86 (*c*Eper2) protein‐coding genes predicted as horizontally transferred between *Cardinium* and other bacteria, including 69, 95, 53, and 56 with a predicted partner organism. Approximately half of all predicted HGT events in each genome were transposase genes, with *c*Eina2 containing over twice as many HGT transposases as the other three genomes, likely because it encodes by far the most predicted transposases (152). As expected, other obligate intracellular bacteria, such as members of the order *Rickettsiales* (*Wolbachia*, *Rickettsia*, and *Midichloria*), made up the majority of predicted HGT partners for *c*Ehis1, *c*Eina2, *c*Eina3, and *c*Eper2.

Some annotated genes were predicted as HGT events in all four genomes, such as a PPDK (EC 2.7.9.1) with the highest similarity to genes from *Midichloria mitochondrii* (*Rickettsiales*) (Sassera et al. 2006), a phosophopyruvate hydratase (enolase) (EC 4.2.1.11) from *Ricketsiella* (*Legionellales*), and an endonuclease III gene from *Rickettsia* (*Rickettsiales*). Type I secretion system components predicted in *c*Ehis1, *c*Eina2, and *c*Eina3 (discussed above) are HGT candidates with *Rickettsia*, suggesting the presence of a nonancestral T1SS in these genomes. Finally, HGTector predicted that biotin synthase genes from *c*Ehis1, *c*Eina2, and *c*Eina3 were horizontally transferred with *Wolbachia*. The cluster of biotin synthesis genes (*bioA*, *bioD*, *bioC*, *bioH*, *bioF*, and *bioB*) from *c*Eina2 and *c*Eina3 shares high amino acid identity (60%–80%) with homologs from *Wolbachia*. The *bio* genes in *c*Eina2, *c*BtQ1 (which is missing *bioB*), and *c*Sfur also occur in the same order as the *bio* gene cassette from *Wolbachia* strain *w*Cle, but this order is reversed in *Cardinium* strains *c*Eper1 and *c*Eina3 (Nikoh et al. 2014; Santos‐Garcia et al. 2014; Zeng et al. 2018). Further, *A. asiaticus*, the closest known relative to *Cardinium*, lacks this biotin synthesis gene cassette, lending more credence to the horizontal transfer of this cassette in *Cardinium*.

**Figure 3:**
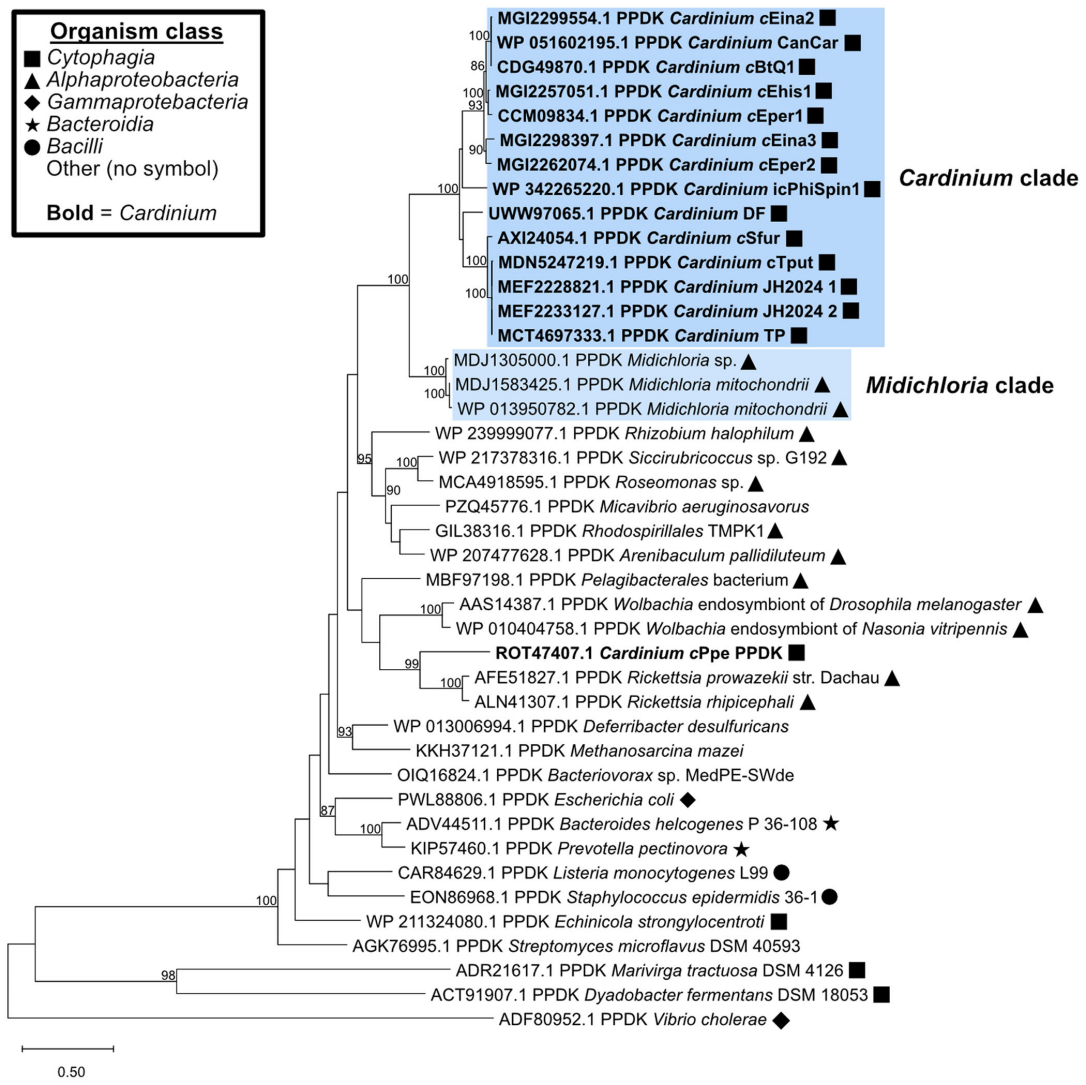
Tree of pyruvate phosphate dikinase (PPDK) amino acid sequences from *Cardinium* compared with other organisms. The tree was built with MEGA12 (Kumar et al. 2024) using the Maximum Likelihood method and Jones–Taylor–Thornton model of amino acid substitutions (Jones et al. 1992) with a total of 1000 bootstrap replicates. The tree with the highest log likelihood (−41,394.33) is shown. Bootstrap confidence values greater than 80 are shown next to each node, and a branch length scale bar representing amino acid substitutions is given in the bottom left of the figure. Protein accessions are given at the beginning, and the class of the organism encoding each protein is shown via a symbol at the end of the entry. Clusters containing *Midichloria‐* and most *Cardinium‐*encoded PPDK proteins are highlighted with light and dark blue backgrounds, respectively.

Other HGT candidates may be important for metabolism in *Encarsia*‐associated *Cardinium*. PPDK (EC 2.7.9.1), an enzyme in the pyrophosphate‐dependent glycolysis pathway responsible for interconverting pyruvate and phosphoenolpyruvate (PEP), is encoded in each of the four newly assembled *Cardinium* genomes and *c*Eper1 but is absent in *A. asiaticus*. This HGT candidate may allow *Cardinium* to generate ATP from PEP, releasing pyruvate for use elsewhere (Minges et al. 2017). A phylogenetic tree of PPDK proteins (Figure 3) revealed that *Cardinium* PPDK proteins (besides strain *c*Ppe, Brown et al. 2018) cluster closely with homologs in *M. mitochondrii* rather than PPDK from other bacteria in the class *Cytophagia*, further supporting its status as a horizontally transferred gene. *M. mitochondrii* is an obligate symbiont of the tick *Ixodes ricinus* and resides in the mitochondria of tick oocytes (Sassera et al. 2006; Uzum et al. 2023). Previous studies have shown that PPDK can be horizontally transferred even between distantly related organisms, including between bacteria and eukaryotes (Slamovits and Keeling 2006; Chastain et al. 2011). Further, *Cardinium* has been previously identified in *Ixodes* ticks (Kurtti et al. 1996), so it is not unlikely that the two symbionts may at least be able to infect the same host organism, leading to opportunities for HGT. The placement of PPDK from the nematode *Cardinium* strain *c*Ppe with those encoded by *Rickettsia* sp. suggests this strain may have obtained this protein independently from other *Cardinium* strains in a separate event. Interestingly, enolase, another core glycolysis enzyme that generates PEP from 2‐phosphoglycerate, was also predicted to be involved in an HGT event between *Cardinium* and *Rickettsiella*. PEP generated from one horizontally transferred core metabolism gene (enolase) may become the substrate for another (PPDK), potentially allowing *Cardinium* to produce energy via two genes it acquired horizontally; however, this has not been experimentally explored. The horizontal transfer of potentially important metabolic enzymes is intriguing, given the limited metabolic capacity of *Cardinium* (see below), so it is possible that HGT may play an important role in the acquisition and maintenance of core metabolic processes in *Cardinium*.

### 3.6 Metabolism and Biosynthetic Capabilities of *Encarsia*‐Associated *Cardinium* Genomes

**Figure 4:**
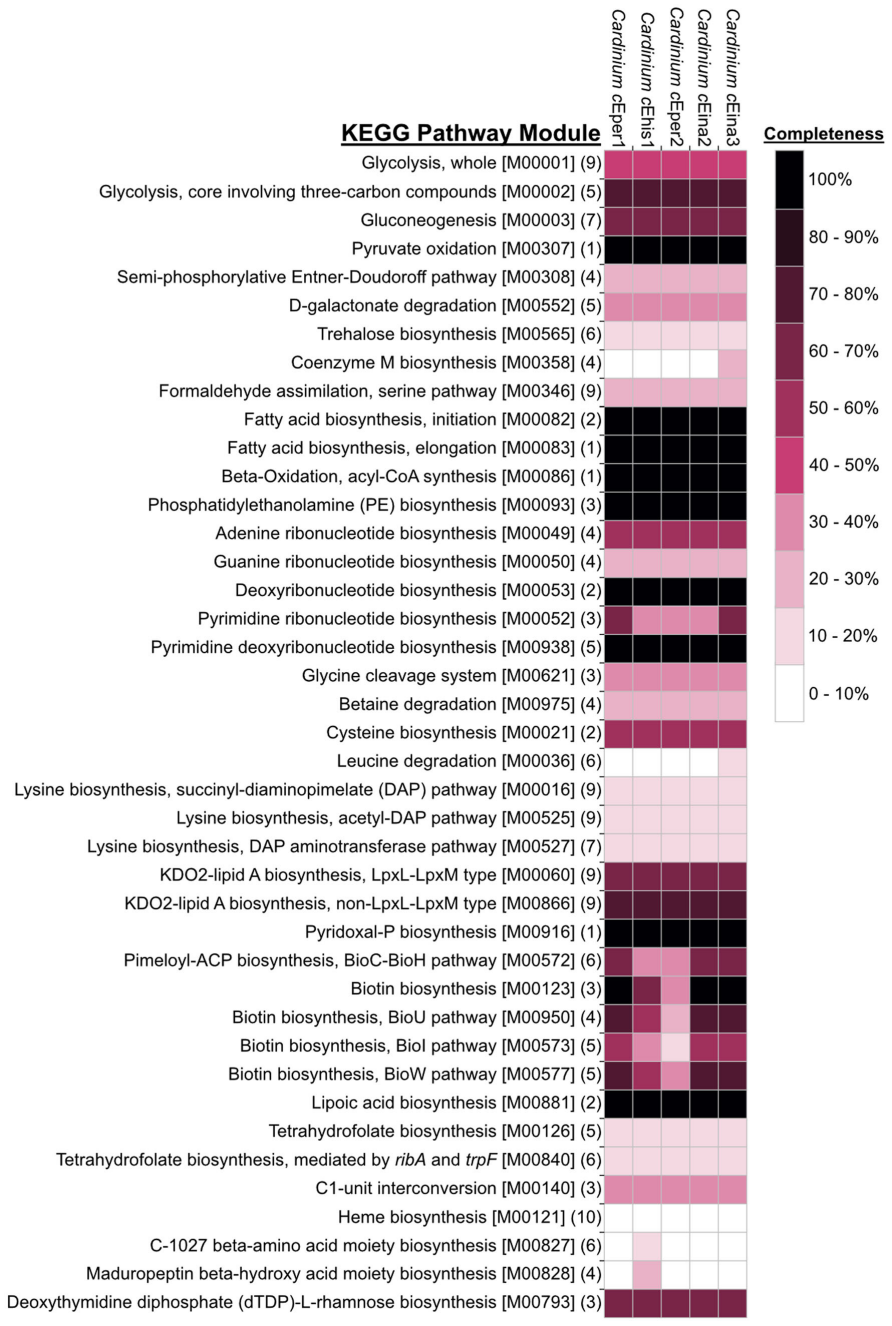
Predicted metabolism of *Encarsia*‐associated *Cardinium* strains. An assessment of the metabolic potential of *Encarsia*‐associated *Cardinium* genomes was generated using the GhostKOALA service from KEGG (Kanehisa et al. 2016) to predict module completeness (i.e., the percentage of genes in a module encoded by each genome), which was then compiled into a heatmap with JColorGrid (Joachimiak et al. 2006). Numbers in parentheses at the end of each pathway name indicate the total number of genes in that pathway, and the accession for each KEGG pathway module is given in brackets. KEGG, Kyoto Encyclopedia of Genes and Genomes.

Similar to other *Cardinium* strains, the newly assembled *Encarsia*‐associated *Cardinium* genomes have very limited biosynthetic and metabolic capacity. Few complete metabolic pathways have been retained, and many features common to free‐living bacteria are absent. Strains *c*Eina2 and *c*Eina3 have retained the greatest pathway completeness among the four genomes assembled in this study, and both have biosynthetic potential similar to *c*Eper1. Generally, *c*Eina2, *c*Eina3, *c*Eper2, and *c*Ehis1 encode similar repertoires of metabolism and biosynthesis, likely due to their relatively close evolutionary distance and shared host genus. However, some key differences exist (Figure 4).

All five *Encarsia*‐associated *Cardinium* genomes encode a partial glycolysis pathway, with only some of the 3‐carbon pay‐off steps being retained, similar to other *Cardinium* (Penz et al. 2012; Santos‐Garcia et al. 2014; Brown et al. 2018; Zeng et al. 2018). Phosphoglycerate mutase and pyruvate kinase appear to be absent, so pyruvate is likely not synthesized via these enzymes. The pyruvate dehydrogenase complex is still present for the conversion of pyruvate to acetyl‐CoA, despite the absence of the tricarboxylic acid cycle. The pentose phosphate pathway is also absent, confirming that central metabolic pathways are significantly degraded in the analyzed genomes. The electron transport chain is also absent in all four genomes. Other bacterial housekeeping genes, such as ATP synthase, tRNAs for all 20 amino acids, and 52 (*c*Ehis1) or 53 (*c*Eina2, *c*Eina3, *c*Eper2) ribosomal proteins, are present. Forty‐three DNA repair and recombination enzymes are encoded in each genome, including *recA*, which may aid in homologous recombination (Penz et al. 2012).

Accessory metabolic and biosynthetic capacity is also limited in these *Cardinium* genomes, but some important pathways remain. Notably, *c*Eina2 and the CI‐causing *c*Eina3 have the complete pathway for biotin biosynthesis, a feature previously described only in the CI‐causing *Cardinium* strains *c*Eper1 and *c*Sfur (Penz et al. 2012; Zeng et al. 2018). Both *c*Eina2 and *c*Eina3 encode the entire core biotin biosynthesis pathway (*bioA*, *bioD*, *bioF*, *bioB*) and some accessory genes (*bioC* and *bioH*) in a cassette of six consecutive genes which may have been involved in an HGT event with *Wolbachia*, as mentioned above. Strain *c*Ehis1 contains a reduced biotin biosynthesis pathway, encoding only *bioB*, *bioF*, and *bioA* in its *bio* gene cassette. Biotin synthesis in *c*Eper2 is the most reduced, with all cassette genes absent besides *bioD* and *bioA*. Strains *c*Eina2 and *c*Eina3 also encode four of the six genes in the biosynthesis pathway for the biotin precursor pimeloyl‐ACP, while *c*Ehis1 and *c*Eper2 only encode two genes in this pathway. Biotin (vitamin B_7_) is an important vitamin for insects, and those with restricted diets are often infected by symbiotic bacteria that can produce B vitamins and provide them to their host (Serrato‐Salas and Gendrin 2022). However, *Encarsia* parasitoid wasps likely acquire biotin from their whitefly diet and are unlikely to require B vitamin provisioning from *Cardinium* to survive, raising questions about the role of this pathway in *Encarsia*‐associated *Cardinium*. It is currently unknown if biotin provisioning by *c*Eper1, *c*Eina2, and *c*Eina3 has an impact on the fitness of their *Encarsia* hosts, although antibiotic curing of *Cardinium* does not show obvious fitness deficits in these species (Perlman et al. 2008; White et al. 2011).

Other metabolites that *c*Ehis1, *c*Eina2, *c*Eina3, and *c*Eper2 can likely synthesize include pyridoxal phosphate and lipoate, which are important cofactors for many enzymatic reactions (Eliot and Kirsch 2004; Spalding and Prigge 2010). Nucleotide biosynthesis is incomplete, and no amino acids can be completely synthesized by any of the four strains. The capacity to synthesize membrane and cell wall components seems to be partially retained. This is evident through the presence of the full biosynthesis pathway for phosphatidylethanolamine, an important membrane phospholipid (Murzyn et al. 2005), as well as most genes involved in the synthesis of dTDP‐l‐rhamnose (precursor to rhamnose, a component of lipopolysaccharide) and some genes in the peptidoglycan synthesis pathway in all four genomes.

## 4 Conclusions

In this study, we assembled and analyzed four *Cardinium* draft genomes for strains that induce CI (*c*Eina3), PI (*c*Ehis1 confirmed, *c*Eper2 unconfirmed), or no discernible reproductive manipulation phenotype (*c*Eina2) in separate species of *Encarsia* parasitoid wasps, more than doubling the total number of available *Cardinium* genomes associated with reproductive manipulation phenotypes. The asymptomatic strain *c*Eina2 was found to be more closely related to *Cardinium* strain *c*BtQ1, a similarly asymptomatic symbiont of *Bemisia* whiteflies, than to other *Encarsia*‐associated *Cardinium*. This suggests the ancestor of *c*Eina2 and *c*BtQ1 may have been horizontally transferred between a parasitoid and the host it parasitizes.

We identified many genes with potential for use by *Encarsia*‐associated *Cardinium* strains to interact with or manipulate their parasitoid wasp hosts. Secretion systems such as the T6SS, common to all *Cardinium*, and the predicted horizontally transferred T1SS in strains *c*Ehis1, *c*Eina2, and *c*Eina3 may play important roles in host manipulation or bacterial competition. Candidate genes for host interaction include homologs of highly expressed genes in the CI‐inducing *Cardinium c*Eper1 and proteins unique to one or more *Encarsia*‐associated *Cardinium* strains with features, such as ankyrin repeats, zinc finger domains, DEAD/DEAH box helicases, and PD‐(D/E)XK nuclease domains. While *c*Eina3 encodes a protein with distant similarity to uncharacterized proteins with PD‐(D/E)XK nuclease domains and a small region of similarity with CidB proteins, no complete homologs to genes implicated in PI or CI caused by *Wolbachia* were found in any of the analyzed genomes of the current study. It is therefore still unclear which factors contribute to the reproductive manipulation phenotypes associated with some of these *Cardinium* strains. Additional functional information is also needed to ascertain how these *Cardinium* strains are manipulating host reproduction and interacting with their hosts, and which roles putative host interaction genes, including those outlined in this study, play in *Cardinium*‐*Encarsia* symbioses.

Our reported *Encarsia*‐associated *Cardinium* genomes are limited in biosynthetic and metabolic potential, but two strains (*c*Eina2 and *c*Eina3) appear capable of producing biotin, a function which was previously only identified in the CI‐causing strains *c*Eper1 and *c*Sfur. Other bacterial symbionts of insects appear to be the most common partners for the horizontal transfer of other protein‐coding genes and TEs with *Cardinium*. Additional core metabolism genes, such as PPDK and enolase, were also predicted to have been horizontally transferred between *Cardinium* and other symbiotic bacteria (*Midichloria* and *Rickettsiella*), suggesting that HGT may play a vital role in the preservation and expansion of metabolism in obligate bacterial symbionts. These genomes will serve as important tools for future *Cardinium* research in areas of reproductive manipulation, host interaction, and beyond.

## Author Contributions

**Dylan L. Schultz:** investigation, conceptualization, formal analysis, software, methodology, visualization, writing – original draft preparation, writing – review and editing. **Corinne M. Stouthamer:** investigation, conceptualization, software, methodology, writing – original draft preparation, writing – review and editing. **Suzanne E. Kelly:** resources, writing – review and editing. **Olivia L. Mathieson:** writing – review and editing. **Manuel Kleiner:** funding acquisition, supervision, writing – review and editing. **Martha S. Hunter:** funding acquisition, conceptualization, supervision, resources, writing – review and editing. **Stephan Schmitz‐Esser:** funding acquisition, conceptualization, data curation, supervision, writing – review and editing.

## Ethics Statement

The authors have nothing to report.

## Conflicts of Interest

The authors declare no conflicts of interest.

## Supporting information


**Figure A1.** Plot of amino acid identities between proteins encoded by *c*Eper1 (outermost ring) to homologs encoded in *c*Ehis1, *c*Eina2, *c*Eina3, and *c*Eper2 (2^nd^ ring to innermost ring, respectively). The plot was generated using the “Proteome Comparison” tool on BV‐BRC and displays the sequence similarities of pairwise BLASTp searches of *c*Eper1 reference proteins against *c*Ehis1, *c*Eina2, *c*Eina3, and *c*Eper2. Blue and green lines indicate high amino acid similarity between the reference *c*Eper1 protein and the homolog identified in the comparison genome, while red and orange lines indicate low similarity.

**File A1.** Synteny comparisons of *Encarsia*‐associated *Cardinium* genomes via dot plots generated by Gepard after reordering contigs with Mauve.

**Table A1.** Accession numbers for proteins similar to highly transcribed cEper1 genes from Table 2.

## Data Availability

Raw sequencing data and genome assemblies have been deposited to NCBI Sequence Read Archive and GenBank under the BioProject PRJNA1257813.
