# Supplementary figures and images for: Comparative Genomics of the Endosymbiont Cardinium Causing Reproductive Manipulation in Encarsia Parasitoid Wasps
Source: Microbiologyopen. 2025 Oct 28;14(6):e70084. doi: 10.1002/mbo3.70084 (PMC12560110; doi:10.1002/mbo3.70084)

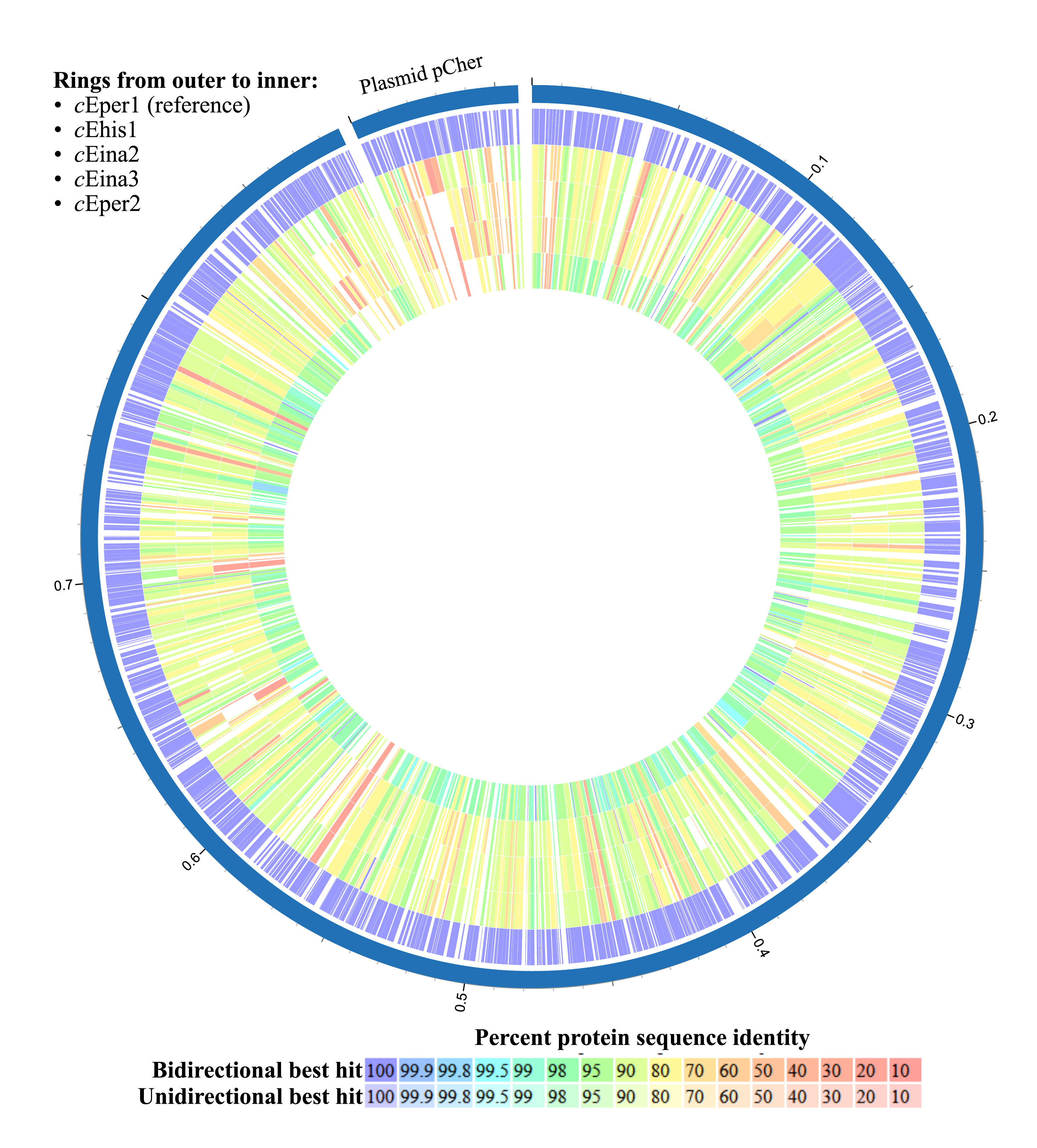

Supplement: Supplementary file 1 — Figure A1. Plot of amino acid identities between proteins encoded by cEper1 (outermost ring) to homologs encoded in cEhis1, cEina2, cEina3, and cEper2 (2nd ring to innermost ring, respectively). The plot was generated using the “Proteome Comparison” tool on BV‐BRC and displays the sequence similarities of pairwise BLASTp searches of cEper1 reference proteins against cEhis1, cEina2, cEina3, and cEper2. Blue and green lines indicate high amino acid similarity between the reference cEper1 protein and the homolog identified in the comparison genome, while red and orange lines indicate low similarity. [file MBO3-14-e70084-s003.jpg]
