# Supplementary material for: Comparative Genomics of the Endosymbiont Cardinium Causing Reproductive Manipulation in Encarsia Parasitoid Wasps
Source: Microbiologyopen. 2025 Oct 28;14(6):e70084. doi: 10.1002/mbo3.70084 (PMC12560110; doi:10.1002/mbo3.70084)

File A1: Synteny comparisons of *Encarsia*-associated *Cardinium* genomes via dot plots generated by Gepard.

X axis: cEper1

Y axis: cEhis1 (contigs reordered to cEper1 using "move contigs" in Mauve)

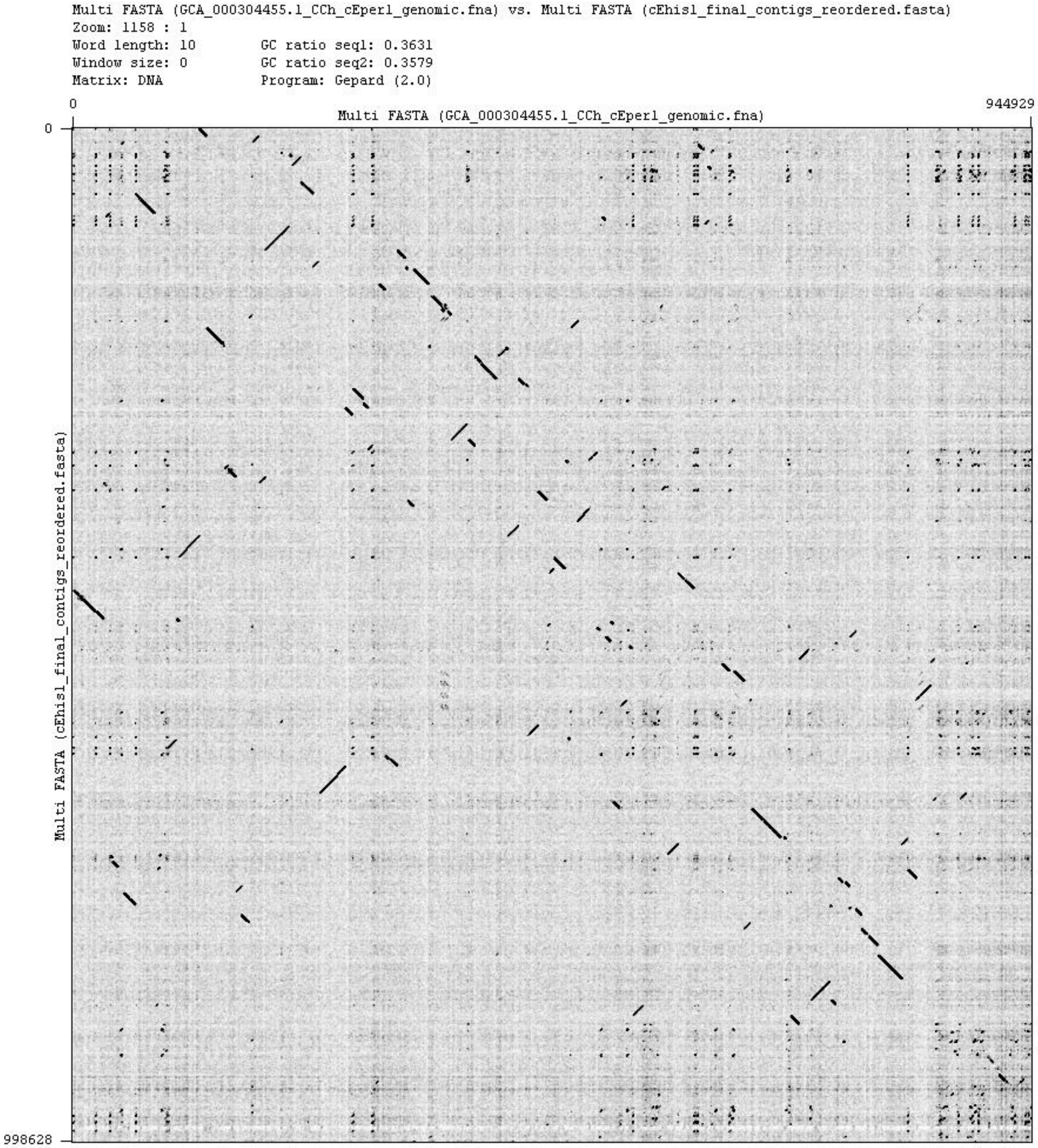

X axis: cEper1

Y axis: cEina2 (reordered to cEper1 using "move contigs" in Mauve)

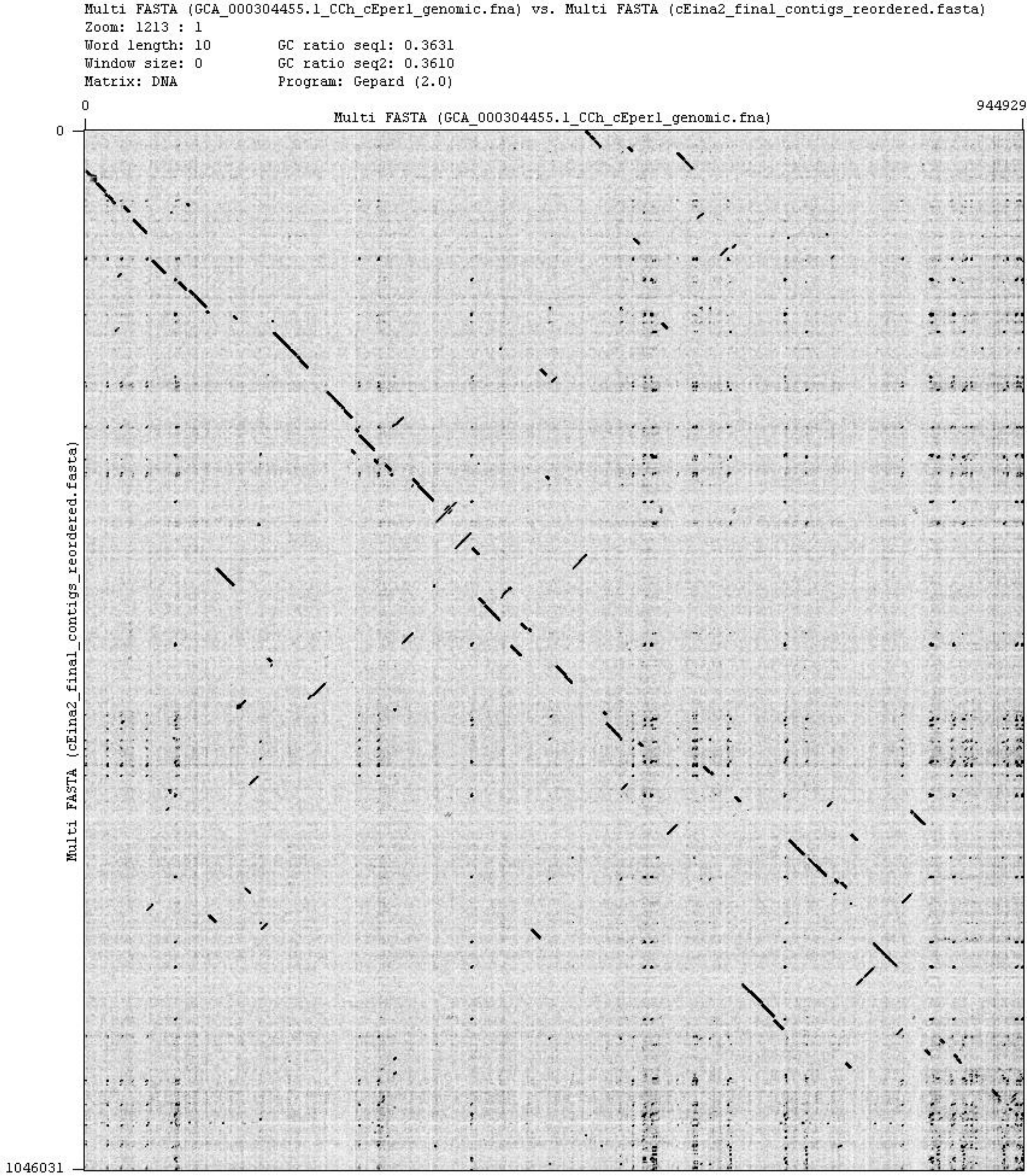

X axis: cEper1

Y axis: cEina3 (reordered to cEper1 using "move contigs" in Mauve)

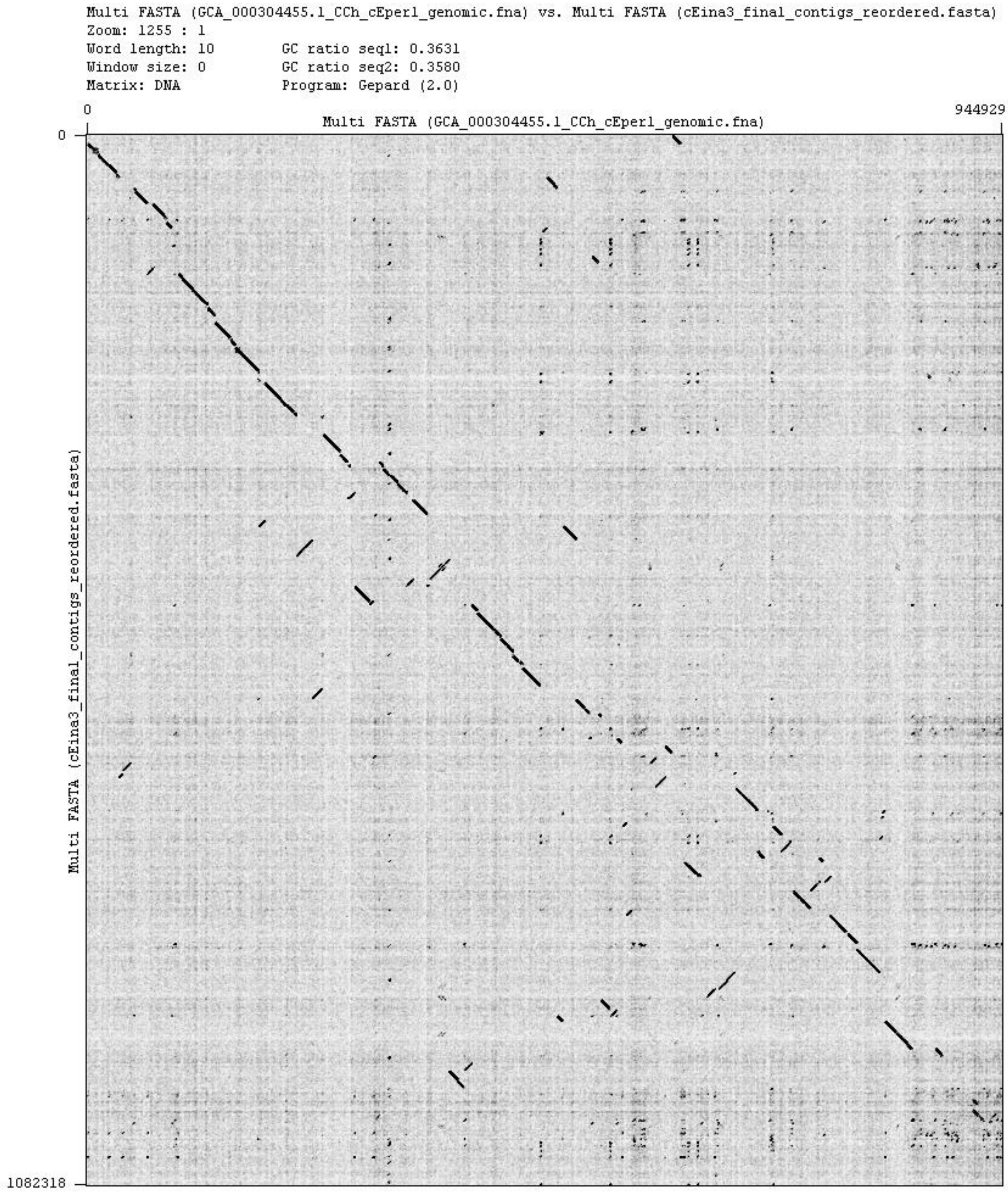

X axis: cEper1

Y axis: cEper2 (reordered to cEper1 using "move contigs" in Mauve)

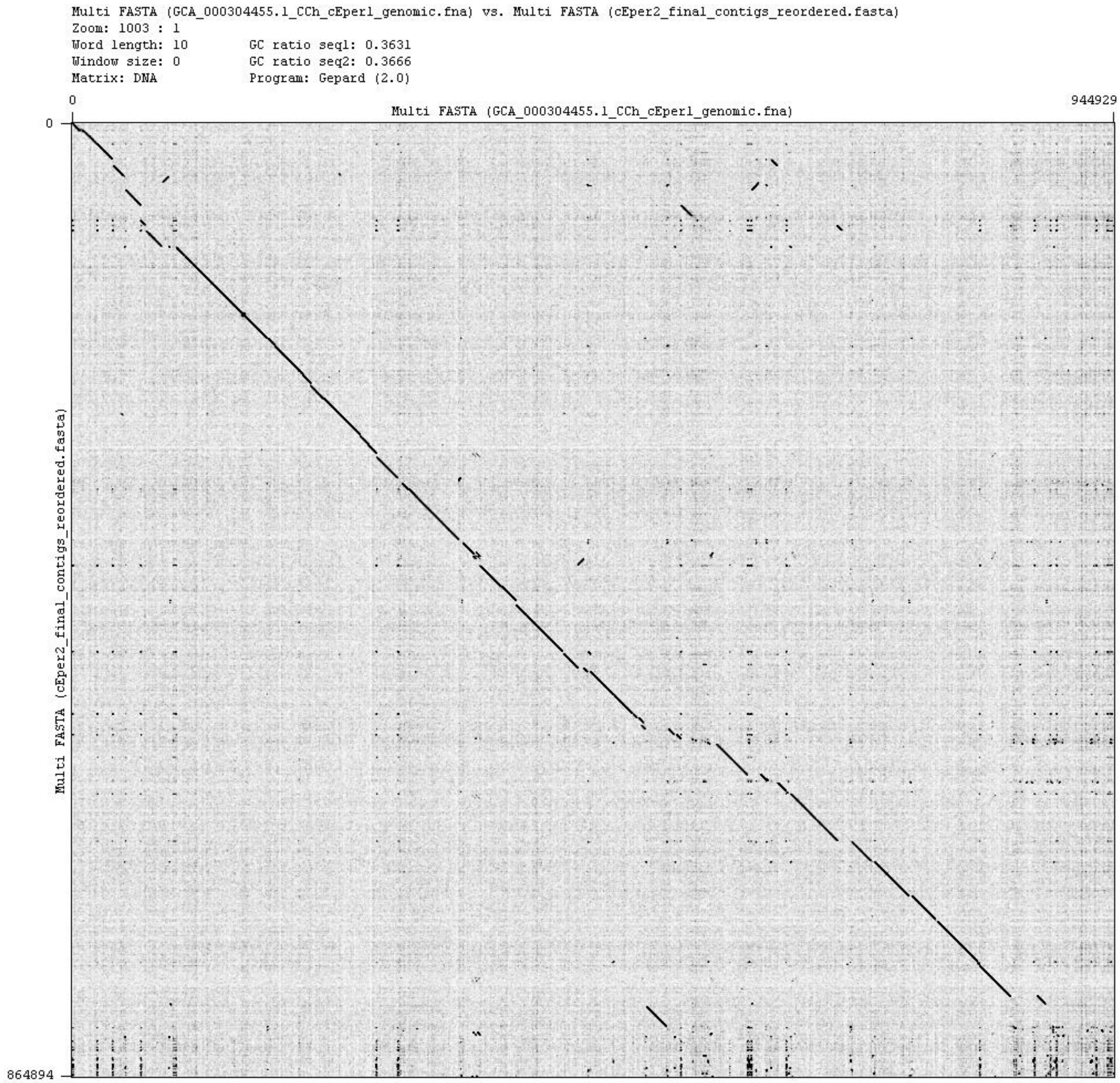

X axis: cEina2 (reordered to cEina3 using "move contigs" in Mauve)

Y axis: cEina3

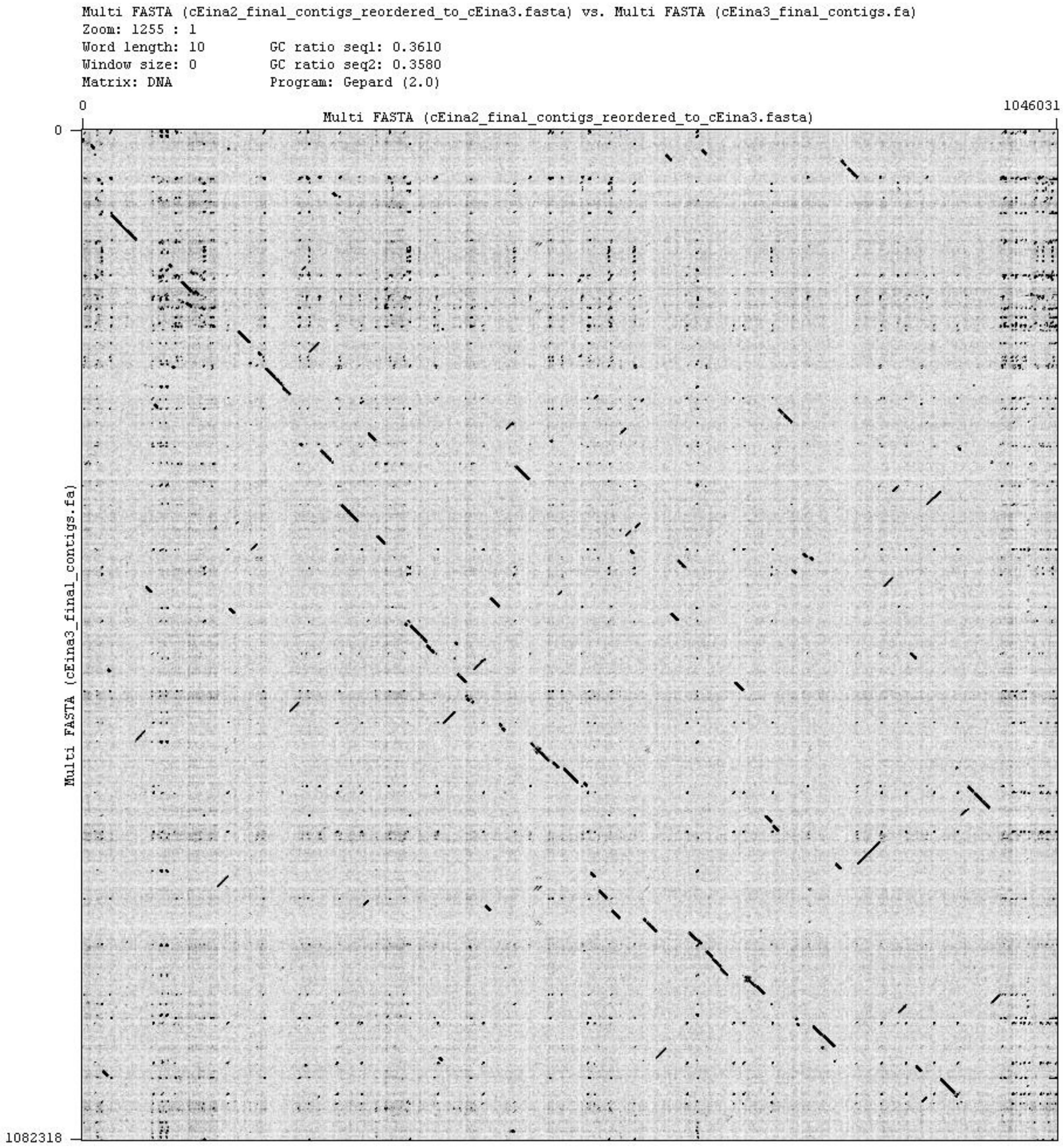

X axis: cEina2 (reordered to cBtQ1 using “move contigs” in Mauve)

Y axis: cBtQ1

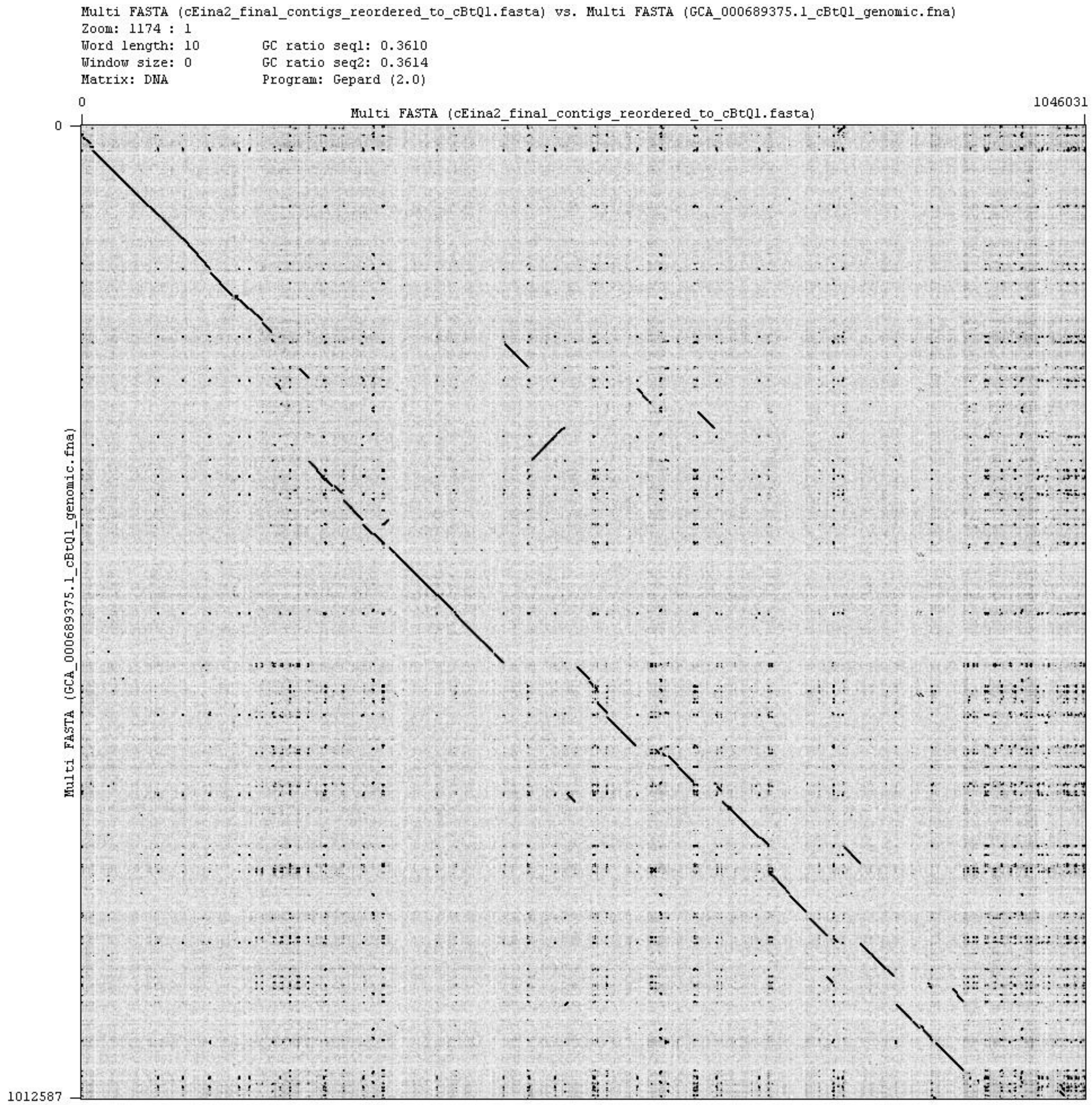

Supplement: Supplementary file 2 — File A1. Synteny comparisons of Encarsia‐associated Cardinium genomes via dot plots generated by Gepard after reordering contigs with Mauve. [file MBO3-14-e70084-s002.pdf]
