# Supplementary material for: Comparative Genomics of the Endosymbiont Cardinium Causing Reproductive Manipulation in Encarsia Parasitoid Wasps
Source: Microbiologyopen. 2025 Oct 28;14(6):e70084. doi: 10.1002/mbo3.70084 (PMC12560110; doi:10.1002/mbo3.70084)
Supplement: Supplementary file 3 — Table A1. Accession numbers for proteins similar to highly transcribed cEper1 genes from Table 2. [file MBO3-14-e70084-s001.docx]

**Table A1.** Accession numbers for proteins similar to highly transcribed *c*Eper1 genes from Table 2. “N/A” indicates there was no hit for the *c*Eper1 protein in the listed genome.

| ***c*Eper1 protein locus tag \| UniProt accession** | **Hit from *c*Ehis1** | **Hit from *c*Eina2** | **Hit from *c*Eina3** | **Hit from *c*Eper2** |
| --- | --- | --- | --- | --- |
| CAHE_0050 \| K0NZZ8 | MGI2257053.1 | MGI2299552.1 | MGI2298399.1 | MGI2262072.1 |
| CAHE_0105 \| K0P009 | MGI2257296.1 | MGI2299267.1 | MGI2298651.1 | MGI2261780.1 |
| CAHE_0390 \| K0P065 | MGI2257437.1 | MGI2299675.1 | MGI2298787.1 | MGI2262093.1 |
| CAHE_0406 \| K0P5U4 | MGI2257422.1 | MGI2299658.1 | MGI2299058.1 | MGI2262105.1 |
| CAHE_0435 \| K0P074 | MGI2257172.1 | MGI2299820.1 | MGI2298602.1 | MGI2262285.1 |
| CAHE_0662 \| K0P2G8 | MGI2257118.1 | MGI2299766.1 | MGI2298976.1 | MGI2262004.1 |
| CAHE_0677 \| K0P2H4 | MGI2257102.1 | MGI2299747.1 | MGI2298579.1 | MGI2262197.1 |
| CAHE_0757 \| K0P2K4 | MGI2257026.1 | MGI2299786.1 | MGI2298239.1 | MGI2261863.1 |
| CAHE_p0007 \| K0PB04 | MGI2257082.1 | MGI2299603.1 | MGI2299055.1 | MGI2262393.1 |
| CAHE_p0014 \| K0P0F9 | MGI2257082.1 | MGI2299603.1 | MGI2299055.1 | MGI2262393.1 |
| CAHE_p0026 \| K0P2Q1 | MGI2257564.1 | N/A | N/A | N/A |
| CAHE_p0043 \| K0P6Z1 | N/A | N/A | MGI2299066.1 | N/A |
